# Frozen-Pair-Type pCCD-Based Methods and Their Double Ionization Variants to Predict Properties of Prototypical BN-Doped Light Emitters

**DOI:** 10.1021/acs.jctc.5c00057

**Published:** 2025-05-14

**Authors:** Ram Dhari Pandey, Matheus Morato F. de Moraes, Katharina Boguslawski, Pawel Tecmer

**Affiliations:** † Institute of Physics, Faculty of Physics, Astronomy, and Informatics, 5170 Nicolaus Copernicus University in Toruń, Grudziadzka 5, 87-100 Toruń, Poland; ‡ Department of Chemistry, 49577 University of Louisville, 2320 S. Brook St. Louisville, Kentucky 40292, United States

## Abstract

Novel, robust, computationally efficient, and reliable theoretical methods are indispensable for the large-scale modeling of desired molecular properties. One such example is the orbital optimized pair coupled-cluster doubles (oo-pCCD) ansatz and its various CC extensions, which range from closed-shell ground- and excited-state models to open-shell variants. Specifically, the ionization-potential equation-of-motion frozen-pair (IP-EOM-fp)­CC methods proved to be competitive with standard CC-type methods for modeling the ionization potentials of organic electronics. In this work, we extend the existing IP-EOM-pCCD-based methods to their double ionization potential (DIP) variants, resulting in various DIP-EOM-fpCC models, including up to double excitations. These methods open the way to reach open-shell singlet, triplet, and quintet states using various pCCD reference functions. Their accuracy is tested for the singlet–triplet gaps of the ortho-, meta-, and para-benzynes. Then, the most accurate models are applied to study the effects of boron and nitrogen doping on designing prototypical naphthalene-based donors and acceptors. Our results demonstrate consistent and reliable outcomes with standard methods and available experimental data. Most importantly, fpCC-type methods show slightly better performance than DIP-EOM-CCSD for strongly-correlated cases and similar performance for systems dominated by dynamical correlation when determining singlet–triplet gaps.

## Graphical abstract

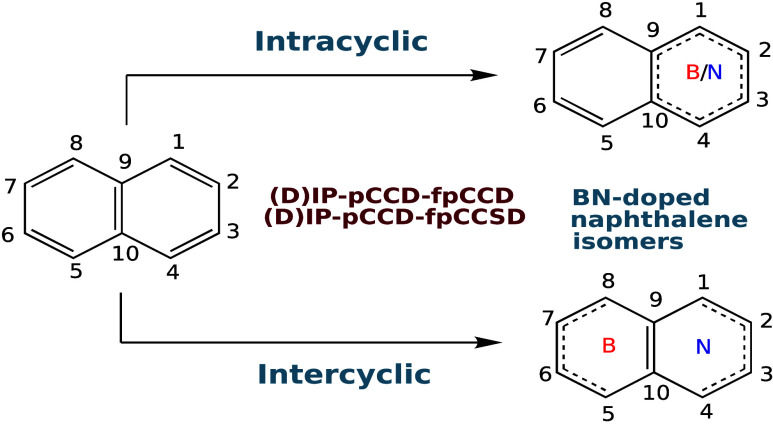

## 1 Introduction

Innovative, reliable, and computationally efficient electronic structure methods are of predominant importance in understanding properties of large molecular structures and building blocks of realistic materials. One promising group of methods that recently emerged is based on the pair Coupled Cluster Doubles^1,2^ (pCCD) reference wave function, often combined with an orbital optimization protocol.^2–7^ Further extensions to pCCD represent promising alternatives to standard electronic structure methods. They include a posteriori perturbation theory,^8,9^ configuration interaction,^10^ and coupled-cluster ground-state energy corrections,^11,12^ linear response theory,^13^ and an equation of motion formalism for electronic excitation energies,^14–16^ ionization potentials (IPs),^17,18^ and electron affinities (EAs).^19^ One research area where these methods seem to be superior is the modeling of electronic structures and properties of large organic molecules, building blocks of organic photovoltaic (OPVs) and organic light-emitting diodes (OLEDs).^13,18–21^ Motivated by the good performance of the frozen-pair variants^11,12^ of the ionization potential equation of motion (IP-EOM-fpCCD and IP-EOM-fpCCSD) and their linearized (L)­CC counterparts (IP-EOM-fpLCCD and IP-EOM-fpLCCSD),^18^ this work elaborates further extensions to double IP (DIP) models. This opens the way to study not only double ionization potentials, but also open-shell singlet and triplet excitation energies and the resulting singlet–triplet gaps. A reliable description of these properties is a cornerstone in understanding and designing modern organic electronic materials with enhanced properties.^22^

One of the most extensively studied organic compounds for OPV materials is polycyclic aromatic hydrocarbons (PAHs).^23–27^ The PAH modification strategies usually incorporate various conjugated units that exhibit either electron-rich (Donor) or electron-deficient (Acceptor) characteristics. By incorporating electron-donating and electron-accepting groups, this approach enables the formation of donor–acceptor (D–A) type structures in various forms, including branched molecules,^28^ helicenes,^29^ polymers, or even more complex arrangements like dendrimers^30^ and supramolecular assemblies. These D and A π-electronic units are fundamental building blocks in determining optoelectronic properties,^31^ including photoluminescence, electroluminescence, quantum efficiency, and energy bandgaps.

Doping is often used to advance and fine-tune organic electronic compounds and their bandgaps.^32–35^ Specifically, integrating boron and nitrogen into organic compounds offers a promising pathway for developing high-performance modern OPVs and other optoelectronic devices.^36,37^ By forming an isoelectronic^38^ pair similar to two carbon atoms, boron and nitrogen atom pairs provide a replacement for carbon–carbon units in polycyclic aromatic hydrocarbons,^39–42^ resulting in BN-embedded or BN-doped π-conjugated systems.^41,43–49^ Altering the BN units’ orientation facilitates electronic structure tuning. As a result, the mono-BN substitution approach can be directed to target each pair of carbon atoms in the naphthalene molecule, leading to the generation of all 23 possible BN-doped isomers. The B–N covalent bond is the most strongly polarized due to the electronegativity difference between boron and nitrogen (opposite the nonpolar C–C covalent bond).^50^ This polarization leads to a charge transfer between neighboring atoms, enhancing intermolecular interactions and the structure’s strength, while leaving planarity, rigidity,^37^ and aromaticity nearly unchanged. Such structural transformation significantly alters the electron distribution within molecules, allowing for a controlled enhancement of optical absorption, HOMO–LUMO gaps, singlet–triplet gaps, chemical reactivity, and charge carrier mobility.^51^

To the best of our knowledge, only a few mono-BN-doped naphthalene electronic structures have been studied to date.^47,52^ The lack of systematic data leads to a significant gap in developing a comprehensive understanding of the CC → BN exchange impact on the electronic structures and the resulting properties. To bridge this gap, this work systematically investigates all possible BN-doped naphthalene molecules with our new DIP-EOM-pCCD-based methodology.

## 2 Theoretical Background

### 2.1 Ground-State pCCD-Based Methods

The pair coupled cluster doubles (pCCD) ansatz^1–4^ is a cost-effective wave function model for describing strongly correlated closed-shell systems

1 $$\left|\mathrm{pCCD}\right\rangle =e^{{\mathrm{T̂}}_{\mathrm{pCCD}}}\left|\Phi_{0}\right\rangle$$

where *T̂*_pCCD_ is a cluster operator containing electron-pair excitations with an overall zero spin

2 $${\mathrm{T̂}}_{\mathrm{pCCD}}=\sum_{i}^{n_{\mathrm{occ}.}}\sum_{a}^{n_{\mathrm{virt}.}}c_{i}^{a}a_{a}^{†}a_{\mathrm{a̅}}^{†}a_{\mathrm{i̅}}a_{i}$$

and |Φ_0_⟩ is some reference determinant (for example, Hartree–Fock). The sum in eq 2 runs over all occupied *i* and virtual *a* orbitals, where *a*_*p*_^†^, *a*_*p̅*_^†^, and *a*_*p*_, *a*_*p̅*_ refer to the electron creation and annihilation operators, and *p* and *p̅* represent spin-up (α) and spin-down (β) electrons, respectively. *c*_*i*_^*a*^ in eq 2 denotes the pCCD cluster amplitudes. The ansatz’s exponential form maintains size-extensivity, while the size-consistency is obtained through variational orbital optimization.^2–5,7^ The resulting pCCD orbitals are localized in nature, enabling us to model quantum states with (quasi)­degeneracies and, thus, strong (static/nondynamical) electron correlation effects.^3,6,20,21,53–56^ The missing weak (dynamic) electron correlation effects that go beyond the electron pairs are covered by a posteriori inclusion of broken pairs. While there are many ways to account for dynamical electron correlation effects on top of the pCCD reference wave function,^8–10,57–59^ coupled-cluster correction proved to be the most reliable.^11,12,60^ Among these coupled-cluster corrections, the frozen-pair coupled cluster (fpCC) and frozen pair linearized CC (fpLCC) models stood as the most robust and accurate.^11,12,56,61^

The fpCC ansatz reads as^12^

3 $$\left|\mathrm{fpCC}\right\rangle =e^{{\mathrm{T̂}}^{\mathrm{ext}}}\left|\mathrm{pCCD}\right\rangle =e^{{\mathrm{T̂}}^{\mathrm{ext}}}e^{{\mathrm{T̂}}_{\mathrm{pCCD}}}\left|\Phi_{0}\right\rangle$$

where the *T̂* ^ext^ cluster operator acts on the pCCD reference and includes electron excitations beyond electron pairs. We consider two forms of the external cluster operator

4 $${\mathrm{T̂}}^{\mathrm{ext}}={\mathrm{T̂}}_{2}^{\prime }={\mathrm{T̂}}_{2}-{\mathrm{T̂}}_{\mathrm{pCCD}}$$

which includes only the broken-pair double excitations, leading to the fpCCD ansatz, and

5 $${\mathrm{T̂}}^{\mathrm{ext}}={\mathrm{T̂}}_{1}+{\mathrm{T̂}}_{2}^{\prime }$$

which additionally incorporates singles, resulting in the fpCCSD ansatz.

The ground-state wave function ansatz of fpLCC reads as^11^

6 $$e^{{\mathrm{T̂}}_{\mathrm{Lext}}}e^{{\mathrm{T̂}}_{\mathrm{pCCD}}}\left|\Phi_{0}\right\rangle \approx \left(1+{\mathrm{T̂}}^{\mathrm{Lext}}\right)\left|\mathrm{pCCD}\right\rangle =\left|\mathrm{fpLCC}\right\rangle$$

where *T̂* ^Lext^ is the linearized cluster operator that contains double excitations (fpLCCD) or single and double excitations (fpLCCSD), excluding electron pair excitations already present in pCCD. In other words, the coupled cluster equations in fpLCC are linear with respect to the nonpair amplitudes *T̂* ^Lext^ while fully accounting for the coupling between all pair- and nonpair amplitudes.

### 2.2 Extension to Excited-States and Open-Shells

Similarly to standard CC methods, we can utilize the equation of motion (EOM) formalism^62,63^ on top of the fpCC and the fpLCC reference (jointly denoted as fp­(L)­CC­(S)­D),

7 $$\left[{Ĥ}_{N},\mathrm{R̂}\right]\left|\mathrm{fp}\left(L\right)\mathrm{CC}\left(S\right)D\right\rangle =\omega \mathrm{R̂}\left|\mathrm{fp}\left(L\right)\mathrm{CC}\left(S\right)D\right\rangle$$

to obtain electronically excited,^14–16,64,65^ spin-flip,^66^ attached,^17,67–69^ ionized,^17,18,70^ doubly attached, and doubly ionized states.^69^ In eq 7, *Ĥ*_*N*_ represents the Hamiltonian in its normal-product form, ω = Δ*E*_*k*_ – Δ*E*_0_ denotes the difference between the ground- and the *k*-th (excited, spin-flip, (doubly) attached, or (doubly) ionized) state associated with a specific form of the linear *R̂* operator

8 $$\left|\Psi_{k}\right\rangle =\mathrm{R̂}\left(k\right)\left|\mathrm{fp}\left(L\right)\mathrm{CC}\left(S\right)D\right\rangle$$

#### 2.2.1 Double IP Frozen-Pair Formalism

In this work, we derive the working equations for the double ionization potential^71,72^ on top of the fp­(L)­CC­(S)­D references, for which the general form of the linear *R̂* operator reads

9 $${\mathrm{R̂}}^{\mathrm{DIP}}=\frac{1}{2}\sum_{\mathrm{ij}}r_{\mathrm{ij}}{â}_{j}{â}_{i}+\frac{1}{6}\sum_{\mathrm{ijka}}r_{\mathrm{ijk}}^{a}{â}_{a}^{†}{â}_{k}{â}_{j}{â}_{i}+...={\mathrm{R̂}}_{2h}+{\mathrm{R̂}}_{3h1p}+...$$

In the above equation, we deliberately omitted the *k*-dependence for clarity. The resulting DIP-EOM-fp­(L)­CC­(S)­D models are denoted as DIP-EOM-fpCCD, DIP-EOM-fpCCSD, DIP-EOM-fpLCCD, and DIP-EOM-fpLCCSD, depending on whether or not the singles (S) are included in the reference frozen pair ansatz and whether or not the linearized (L)­CC variant is considered. The diagrammatic representation of these models is depicted in Figure 1, more details are also provided in the Supporting Information (SI), including the algebraic form of the corresponding DIP-EOM equations.

**Figure 1:**
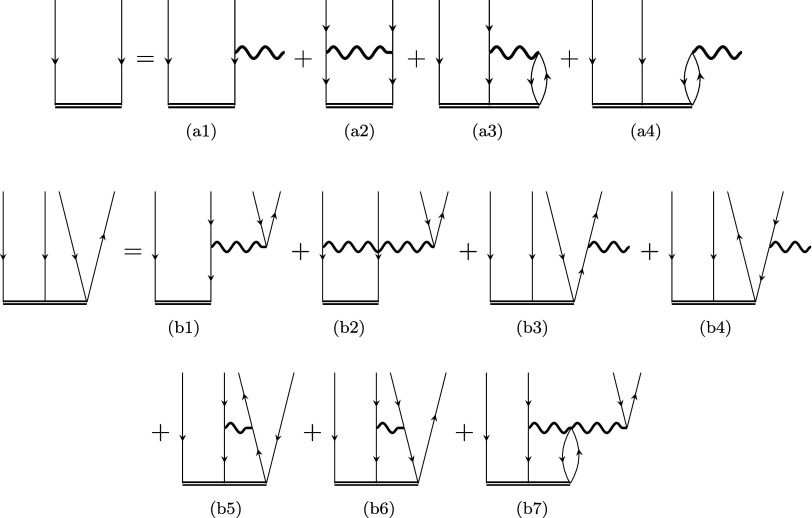
Diagrammatic form of the DIP-EOM-(fp­(L))­CC equations in the antisymmetrized formalism. The algebraic expressions for various fpCC models are summarized in the SI.

In our current implementation, *R̂* ^DIP^ in eq 9 is restricted to 2 holes (2h) and 3 holes and 1 particle (3h1p) operators. Since our Hamiltonian does not include electron spin interactions (such as spin–orbit coupling), we can independently optimize the cases for the following spin projection manifolds: *S*_*z*_ = 0, *S*_*z*_ = −1, and *S*_*z*_ = −2. This typically holds since the electronic Hamiltonian does not contain any information on the electron spin (like spin–orbit coupling) and we can optimize the different spin-projections separately.^73^ In this work, we focus on *S*_*z*_ = 0 states, which allows us to target singlet, triplet, and quintet states within one EOM optimization. The corresponding operator *R̂* ^DIP^ for *S*_*z*_ = 0 states reads

10 $${\mathrm{R̂}}^{S_{z}=0}=\sum_{i\mathrm{j̅}}r_{i\mathrm{j̅}}\hat{\mathrm{j̅}}î+\frac{1}{2}\sum_{i\mathrm{j̅}\mathrm{ka}}r_{i\mathrm{j̅}k}^{a}{â}^{†}\mathrm{k̂}\hat{\mathrm{j̅}}î+\frac{1}{2}\sum_{i\mathrm{j̅}\mathrm{k̅}\mathrm{a̅}}r_{i\mathrm{j̅}\mathrm{k̅}}^{\mathrm{a̅}}{\hat{\mathrm{a̅}}}^{†}\hat{\mathrm{k̅}}\hat{\mathrm{j̅}}î$$

The associated configurational subspace utilized in the diagonalization of the matrix representation is consequently defined by the span of Slater determinants |Φ_*i j̅*_⟩, |Φ_*i j̅k*_^*a*^⟩, and |Φ_*i j̅ k̅*_^*a̅*^⟩ for *N* – 2 electrons. Note that *R̂* ^*S*_*z*_=0^ within the fp­(L)­CC­(S)­D formalism can generate singlet, triplet, and quintet states, *R̂* ^*S*_*z*_=–1^ triplet and quintet states, and *R̂* ^*S*_*z*_=–2^ only quintet states.

### Computational Scaling

The ((D)­IP-EOM-)­pCCD approach is a cost-effective simplification of ((D)­IP-EOM-)­CCD or ((D)­IP-EOM-)­CCSD. The computational cost of IP-EOM-pCCD scales as $O(o^{3}\upsilon^{2})$ , while the DIP variant of EOM-pCCD increases the cost to $O(o^{2}\upsilon^{4})$ . For comparison, the computational bottleneck of (D)­IP-EOM-CCD or (D)­IP-EOM-CCSD is given by the ground-state calculation of $O(o^{2}\upsilon^{4})$ cost, while CCSD­(T) formally scales as $O(N^{7})$ (*N* = *o* + υ). All frozen-pair variants feature a similar computational complexity as their conventional CC counterparts, that is, (D)­IP-EOM-fp­(L)­CC are limited by their corresponding ground-state calculation.

## 3 Computational Details

### 3.1 Calculations Details

All discussed pCCD-based methods above are efficiently implemented in the PyBEST software package,^74,75^ which makes use of the newest NVIDIA GPU architectures and Python external libraries.^76^ All electronic structure calculations with pCCD-based methods^2^ and DIP-EOM-CCSD were performed using a developer version (v2.1.0-dev0) of the PyBEST^74,75^ software package. The 1s orbitals for C, N, and B were kept frozen for all calculations, and the correlation consistent polarized valence double-ζ (cc-pVDZ) and triple-ζ (cc-pVTZ) basis sets were employed.^77^ We used the variational orbital optimization protocol in all pCCD calculations, where the final orbitals are ordered according to the pCCD natural occupation numbers.^3,7^ These orbitals were used for subsequent fp­(L)­CC­(S)­D calculations, including their EOM extensions to compute IPs and DIPs. The electron affinities (EAs) and electronic excitations (EEs) were calculated using the DIP-EOM-fpCC­(S)­D formalism for the double-anion reference wave function,

11 $$E^{\mathrm{EA}}=E_{0}^{\mathrm{IP}}\left[2h,1p\right]-E_{0}^{\mathrm{DIP}}\left[3h,1p\right]$$

12 $$E_{i}^{\mathrm{EE}}=E_{0}^{\mathrm{DIP}}\left[3h,1p\right]-E_{i}^{\mathrm{DIP}}\left[3h,1p\right]$$

where *E*_*i*_[*Xh, Yp*] is the *i*-th root obtained from the *X* holes and *Y* particles EOM formalism. The standard electronic structure calculations were carried out in the Molpro2020 ^78–80^ software package. Additionally, IPs were also evaluated using various computational methods, such as CCSD (coupled cluster singles doubles) and CCSD­(T) (coupled cluster with single, double, and perturbative triple excitations). Similar to the pCCD methodologies, a frozen core was utilized in all conventional calculations and a cc-pVDZ basis set was employed.

### 3.2 Molecular Structures

In this work, we explored three sets of aromatic molecules: benzyne, benzene, and naphthalene. The first set consists of the three benzyne molecules, namely ortho-, meta-, and para-benzynes, which are depicted in Figure 2. The second set focuses on benzene and its mono-, di-, and tri-BN-doped structures (see Figure S1 in the SI). Finally, the third set comprises naphthalene (see Figure 3) along with its 23 mono-BN-doped derivatives (see Figure 4). The geometries for the benzyne molecules were taken from the literature.^82^ For other systems, we considered two types of structures: fully and partially relaxed for the neutral benzene, naphthalene, and its BN-doped variants. The fully relaxed structures were optimized using density functional theory (DFT) as implemented in the Turbomole V7.3 software package,^83,84^ with the BP86 ^85,86^ exchange–correlational functional and the def2-TZVP basis set.^87^ Additionally, we conducted vibrational frequency calculations of the fully relaxed structures to confirm that all optimized structures were energy minima with no imaginary frequencies. The partially relaxed structures of the nondoped systems (benzene and naphthalene) were optimized at the CCSD level of theory using the cc-pVDZ basis set.

**Figure 2:**
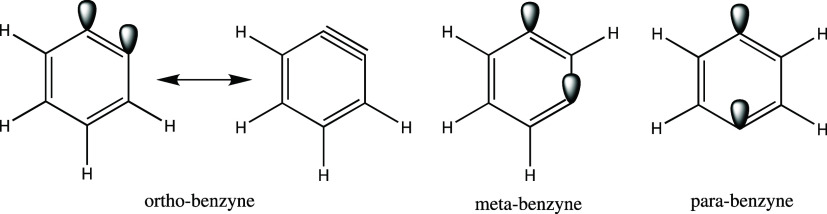
Ortho-, meta-, and para-benzynes in three distinct configurations. The molecules were drawn using the ChemDraw software.^81^.

**Figure 3:**
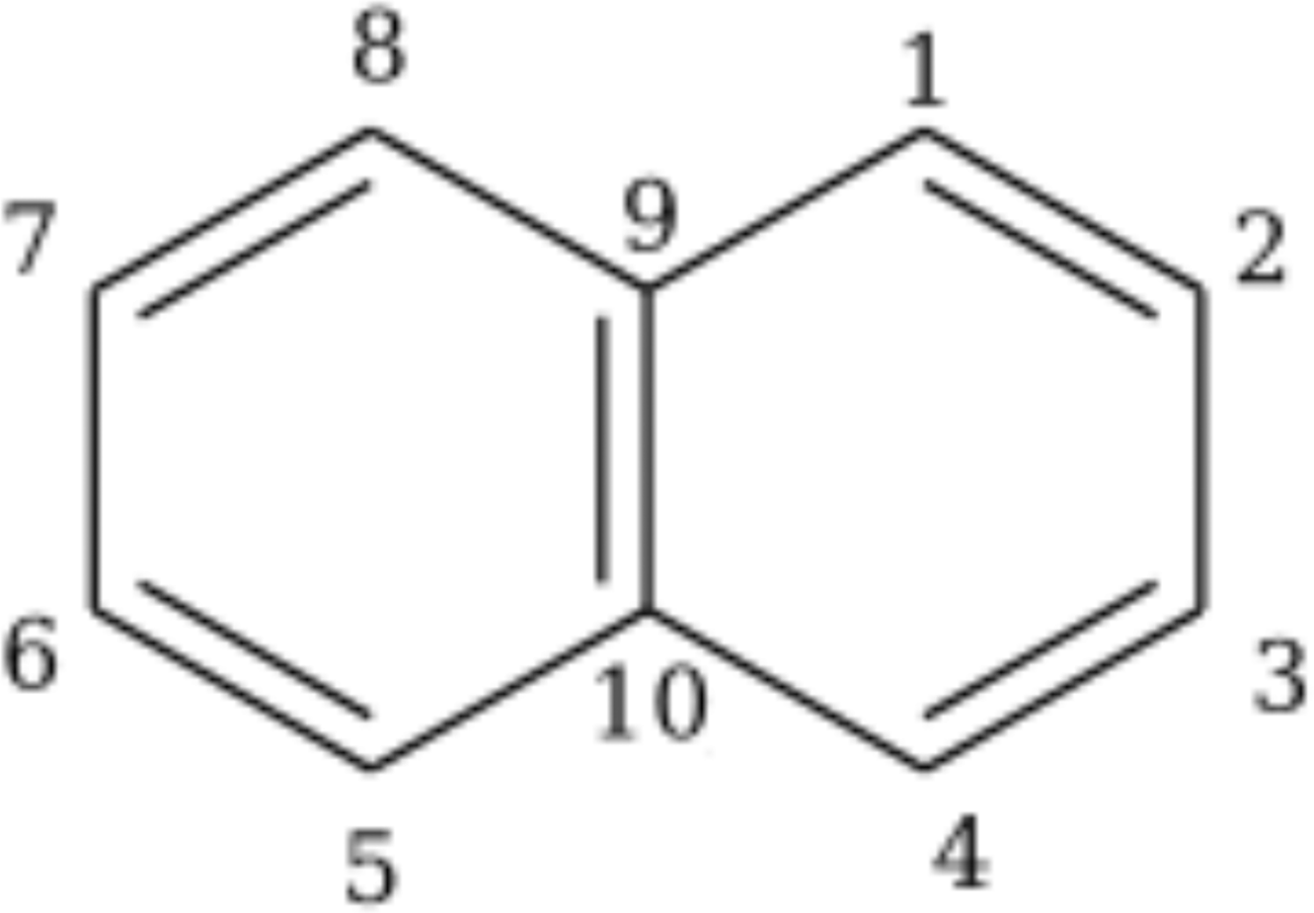
The specific sites of each carbon in the naphthalene ring system.

**Figure 4:**
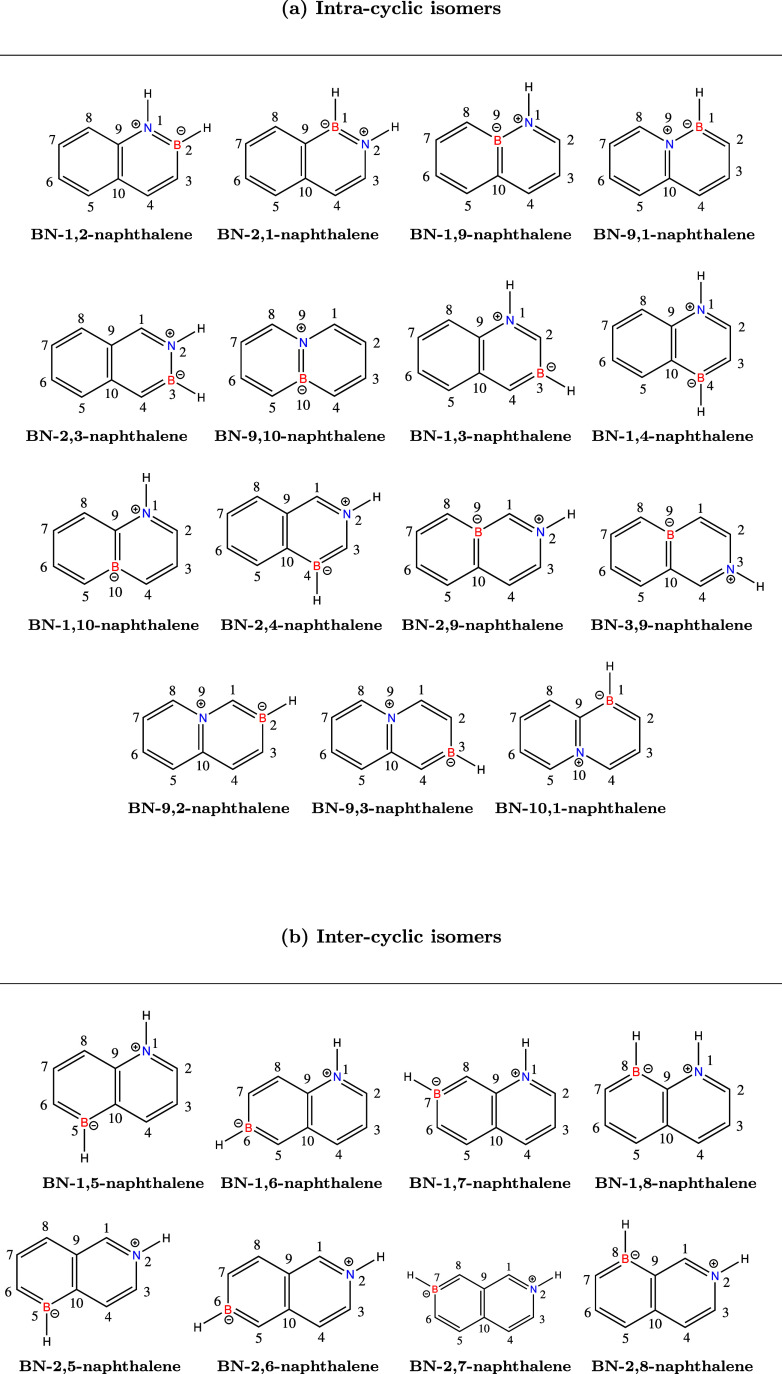
Mono-BN-doped naphthalene isomers with the boron and nitrogen incorporated as intracyclic heteroatoms (a) and as intercyclic heteroatoms (b). The molecules were drawn using the ChemDraw software.^81^

To generate the mono-BN-doped variants, a pair of carbon atoms was replaced with nitrogen and boron without performing further reoptimization. The resulting molecular structures were used in subsequent wave function calculations. The *xyz* coordinates are collected in the SI.

To avoid repetitive labeling of the BN-doped naphthalene isomers, we adopted a specific numbering system mentioned in the literature,^47^ as demonstrated in Figure 4. The first digit indicates the position of the nitrogen atom and the second the position of the boron atom, as indicated in Figure 4. For example, the notation BN-1,2-naphthalene indicates nitrogen at position 1 and boron at position 2. These isomers are divided into two categories: intra-cyclic, where both boron and nitrogen are incorporated within a single ring (Figure 4a), and inter-cyclic (Figure 4b), where the heteroatoms are placed across two different rings.

Six isomers exhibit a BN nearest-neighbor arrangement, where boron and nitrogen are directly adjacent without intervening carbon atoms. These include the BN-1,2-naphthalene, BN-2,1-naphthalene, BN-1,9-naphthalene, BN-9,1-naphthalene, BN-2,3-naphthalene, and BN-9,10-naphthalene isomers (Figure 4a). Seven isomers are characterized by a second nearest-neighbor alignment, where one carbon atom links boron and nitrogen. These are the BN-1,3-naphthalene, BN-1,8-naphthalene, BN-1,10-naphthalene, BN-2,4-naphthalene, BN-2,9-naphthalene, BN-9,2-naphthalene, and BN-10,1-naphthalene systems (Figure 4a,b). Six isomers feature a BN-1,4-like structural arrangement in which two carbon atoms separate the boron and nitrogen atoms. These isomers are identified as BN-1,4-naphthalene, BN-1,5-naphthalene, BN-1,7-naphthalene, BN-2,8-naphthalene, BN-9,3-naphthalene, and BN-3,9-naphthalene (Figure 4a and 4b). Furthermore, three isomers exhibit a BN-1,6-like pattern, where the minimum distance between boron and nitrogen atoms is three carbon atoms, including BN-1,6-naphthalene, BN-2,5-naphthalene, and BN-2,7-naphthalene (Figure 4b). Finally, one specific isomer features a BN pair in a 2,6-like relationship, separating the boron and nitrogen atoms by four intervening carbon atoms (Figure 4b).

## 4 DIP-EOM-fpCC Methods and Their Application to BN-Doped Naphthalene Derivatives

In the following, we will explore four molecular properties: IPs, EAs, singlet–singlet gaps, and singlet–triplet gaps. These properties are accessible through combinations of IP- and DIP-EOM-fpCCSD-based methods as described in Section 3.1 (see eqs 11 and 12). To that end, we start by benchmarking our newly proposed DIP models for singlet–triplet energy gaps of benzynes. Based on this benchmark study, we will focus on the most promising models to investigate the properties of mono-BN-doped naphthalene isomers depicted in Figure 4.

### 4.1 A Benchmark Case for DIP-EOM-fp­(L)­CC­(S)­D: Ortho-, Meta-, and Para-Benzynes

The relative energies of the singlet and triplet states of ortho-, meta-, and para-benzynes (depicted in Figure 2) provide a common testing ground for new quantum chemistry methods.^82,88,89,90,91,92,93,94,95,96^ Unfortunately, a direct comparison between these methods is not straightforward due to different geometries and CC ansätze (single-reference vs multireference) used to study these systems. Accurately modeling the lowest-lying triplet states of benzynes poses a significant obstacle in quantum chemistry because of their distinctive diradical nature. This phenomenon is demonstrated by the strong pairing between the two neighboring radical centers in ortho-benzyne, which brings this isomer near to forming a triple bond. However, in meta- and para-benzyne, the separation between the radical centers increases (as shown in Figure 2), enhancing the diradical character (and the multireference nature) from ortho to meta to para.^82,96^ For the ortho and meta isomers, the singlet and triplet states are of ^1^ *A*_1_ and ^3^ *B*_2_ symmetry (*C*_2*v*_), respectively. In contrast, the associated states for para-benzyne are ^1^ *A*_*g*_ and ^3^ *B*_1*u*_ (*D*_2*h*_). An increase in the biradical character in the ortho → meta → para order and potential orbital instabilities in the singlet state of the para isomer make the benzyne series a challenging test case. To that end, this set is a valid test case to benchmark our newly developed DIP-fp­(L)­CC­(S)­D models, keeping in mind potential problems related to the unstable dianion reference.^97,98^

Table 1 provides a summary of the adiabatic singlet–triplet energy gaps computed using DIP-EOM-fpCC methods (DIP-EOM-fpCCD and DIP-EOM-fpCCSD) and their linearized (L)­CC variants (DIP-EOM-fpLCCD and DIP-EOM-fpLCCSD) employing the cc-pVDZ basis set. We should stress here that the molecular structures of all benzyne isomers were optimized for a double-ζ-quality basis.^82^ We performed additional numerical tests using a cc-pVTZ basis set and the double-ζ-optimized molecular structures. Our calculations suggest that the triple-ζ results do not correspond to the adiabatic singlet–triplet gaps, where the vertical excitation energies are approximately 0.1 eV lower than the adiabatic ones. A more detailed analysis of the influence of molecular structure and basis set size on the singlet–triplet gaps of the benzyne isomers is presented in the SI. Therefore, we scrutinize the cc-pVDZ results below, while the cc-pVTZ results are summarized in the SI. The overall performance of DIP-EOM-pCCD and DIP-EOM-fp­(L)­CC­(S)­D methods is very good. The singlet-triplet energy for all models and basis sets diminishes with the increasing separation between the two radicals. The only exception is the DIP-fpLCCSD/cc-pVTZ result for ortho-benzene, for which we encountered convergence difficulties for the ^1^ *A*_1_ reference state. The studied fpCC methods and the corresponding LCC variants predict the correct order of states and provide results comparable with experimental data (see the bottom row in Table 1 denoted as “Experimental-ΔZPE”) and CCSD­(T) results. We should stress here that not all the methods listed in Tables 1 and S1 predict the correct sign for the singlet–triplet gap of para-benzyne when the canonical Hartree-Fock orbitals are utilized.^94^ This underlines the need for the use of natural pCCD orbitals in the reference state for our DIP-EOM-fp­(L)­CC­(S)­D models.

**Table 1:** Total Ground-State Energies (in *E*_h_) and Adiabatic Excitation Energies (in eV) of the Lowest-Lying Triplet States of Ortho-, Meta-, and Para-Benzynes Using the cc-pVDZ Basis Set

	ortho-benzyne		meta-benzyne		para-benzyne	
methods	^1^ *A*_1_	^3^ *B*_2_	^1^ *A*_1_	^3^ *B*_2_	^1^ *A*_*g*_	^3^ *B*_1*u*_
DIP-EOM-pCCD	–229.745342	1.489	–229.733659	0.930	–229.712344	0.217
DIP-EOM-fpCCD	–230.165536	1.604	–230.149675	0.712	–230.142487	0.164
DIP-EOM-fpLCCD	–230.190942	1.585	–230.175352	0.688	–230.169755	0.171
DIP-EOM-fpCCSD	–230.165536	1.529	–230.167839	0.741	–230.156895	0.131
DIP-EOM-fpLCCSD	–230.221424	1.671	–230.201990	0.719	–230.192802	0.051
DIP-EOM-CCSD	–230.191019	1.601	–230.175684	0.749	–230.162584	0.190
CCSD^99^ ^,^ ^a^	–230.2184	1.214	–230.193434	0.466	–230.154084	–0.723
CCSD(T)^99^ ^,^ ^a^	–230.26247	1.434	–230.239179	0.772	–230.219135	0.071
experimental^100,101^		1.628 ± 0.013		0.911 ± 0.014		0.165 ± 0.016
ΔZPE^82^		–0.028		0.043		0.021
experimental-ΔZPE		1.656		0.868		0.144

a CCSD and CCSD­(T) refer to energy differences for state-specific calculations (singlet and triplet state) using an older cc-pVDZ basis set and slightly different geometries (see ref 99).

However, the errors with respect to experimental values are significantly smaller for DIP-EOM-fp­(L)­CC­(S)­D/cc-pVDZ compared to conventional CCSD/cc-pVDZ and CCSD­(T)/cc-pVDZ. Except of DIP-EOM-fpLCCSD, our fpCC methods provide similar results to DIP-EOM-CCSD (using canonical HF orbitals) for ortho- and meta-benzynes, and are closer to experiment when strong correlation becomes important (the para-benzyne case). While DIP-EOM-fpLCCSD predicts singlet–triplet gaps closest to the refernce for some systems, DIP-EOM-fpCCD provides the most consistent results across the whole series.

In general, the cc-pVDZ results agree better with the experimental reference gaps. Specifically, the cc-pVTZ singlet–triplet energy gaps deviate from “Experimental-ΔZPE” measurements by 0.01–0.19 eV. The smallest errors are associated with ortho- and meta-benzynes (0.01–0.12 eV), whereas para-benzyne features larger discrepancies, lying between 0.16 eV (3.6 kcal/mol) and 0.19 eV (4.3 kcal/mol). Most likely, this is due to the chosen molecular structures (see SI for more details and Table S2 of the SI for the vertical excitation energies). Most importantly, the proposed methodologies outperform the conventional CCSD approach while performing slightly better than the conventional DIP-EOM-CCSD variant. To conclude, our initial assessment suggests that the DIP-EOM-fpCC­(S)­D/cc-pVDZ models should be used as a reference for further predictions, especially in cases when the strong correlation is important.

### 4.2 Experimental and Theoretical Comparison of BN-Doped Naphthalene Isomer Properties

Our first objective is to ensure reliable predictions of IPs for the investigated mono-BN-doped naphthalene isomers using fpCC­(S)­D-based methods. To assess their accuracy, we compare the predicted IPs of our fpCC­(S)­D formalism with experimental reference data of five molecules from the literature, which includes naphthalene and its four mono-BN-doped naphthalene isomers and the CCSD­(T) results of all mono-BN-doped isomers.

To the best of our knowledge, this is the largest collection of experimental data measured by UV-photoelectron spectroscopy,^47,102,103,104,105^ enabling a direct comparison with the golden standard state-specific CCSD­(T) method. Table 2 presents a comparative statistical analysis of theoretically predicted IPs using conventional and frozen-pair methods, where the mean signed error (MSE) and the root mean square error (RMSE) are evaluated for fully relaxed geometries with respect to the experimental values and the CCSD­(T) results.

**Table 2:** Calculated Ionization Potentials (IPs) in eV of Naphthalene and 23 Mono-BN-doped Naphthalene Isomers Using Both Conventional and pCCD-Based Approaches with the cc-pVDZ Basis Set^a^

	fully relaxed geometry				
	standard methods		IP-EOM		
molecule	CCSD	CCSD(T)	fpCCD	fpCCSD	exp.
naphthalene	7.94	7.92	8.14	8.04	8.14 ^105^
BN-1,2-naphthalene	8.09	8.12	8.11	8.10	8.45 ^47^
BN-1,3-naphthalene	7.29	7.37	7.57	7.50	
BN-1,4-naphthalene	7.81	7.79	7.86	7.84	
BN-1,5-naphthalene	6.68	6.75	6.82	6.81	
BN-1,6-naphthalene	6.68	6.82	6.81	6.82	
BN-1,7-naphthalene	7.14	7.20	7.27	7.26	
BN-1,8-naphthalene	6.58	6.69	6.64	6.68	
BN-1,9-naphthalene	7.48	7.53	7.74	7.68	7.78 ^47^
BN-1,10-naphthalene	7.31	7.40	7.47	7.45	
BN-2,1-naphthalene	7.68	7.69	7.86	7.79	
BN-2,3-naphthalene	7.19	7.25	7.35	7.31	
BN-2,4-naphthalene	7.05	7.15	7.33	7.27	
BN-2,5-naphthalene	6.40	6.51	6.66	6.62	
BN-2,6-naphthalene	7.08	7.14	7.25	7.23	
BN-2,7-naphthalene	6.61	6.75	6.69	6.70	
BN-2,8-naphthalene	6.68	6.74	6.88	6.85	
BN-2,9-naphthalene	7.42	7.49	7.58	7.54	
BN-3,9-naphthalene	7.47	7.50	7.73	7.68	
BN-9,1-naphthalene	7.10	7.15	7.25	7.22	7.44 ^47^
BN-9,2-naphthalene	7.18	7.29	7.38	7.35	
BN-9,3-naphthalene	7.43	7.48	7.57	7.56	
BN-10,1-naphthalene	6.92	7.01	7.15	7.10	
BN-9,10-naphthalene	8.15	8.08	8.22	8.22	8.42 ^47^
MSE^b^ (exp.)	–0.29	–0.29	–0.15	–0.19	
RMSE^c^ (exp.)	0.30	0.29	0.20	0.21	
MSE^b^ (CCSD(T))	–0.06		0.10	0.07	
RMSE^c^ (CCSD(T))	0.08		0.13	0.09	

a Mean signed error (MSE) and root mean square error (RMSE) with respect to the experiment and CCSD­(T) results.

b $\mathrm{MSE}=\frac{1}{N}\sum_{i}^{N}\left(E_{i}^{\mathrm{method}}-E_{i}^{\mathrm{ref}}\right)$ .

c $\mathrm{RMSE}=\sqrt{\sum_{i}^{N}\frac{{\left(E_{i}^{\mathrm{method}}-E_{i}^{\mathrm{ref}}\right)}^{2}}{N}}$ .

The frozen-pair (fp)­CC formalism shows error margins relative to the experimental data, ranging from −0.15 to 0.20 eV for IP-EOM-fpCCD and from −0.19 to 0.21 eV for IP-EOM-fpCCSD. However, for conventional methods, such as CCSD and CCSD­(T), deviation in errors ranges from −0.29 to 0.30 and −0.29 to 0.29, respectively.

CCSD and CCSD­(T) methods underestimate the experimental ionization potentials, as indicated by their identical MSE of −0.29 eV. However, their RMSE values overestimate and differ slightly, with values of 0.30 and 0.29 eV, respectively. The frozen-pair approaches (IP-EOM-fpCCD and IP-EOM-fpCCSD) significantly mitigate underestimation, resulting in improved accuracy with experimental values, reducing MSE around 49% (−0.15 eV) for the former and 35% (−0.19 eV) for the latter, as compared to conventional methods. This improvement is further supported by lower RMSE values of 0.20 and 0.21 eV compared to the RMSE values of CCSD and CCSD­(T), suggesting better accuracy. Relative to CCSD­(T), the computed MSE and RMSE for CCSD are −0.06 and 0.08 eV, respectively. However, these errors for frozen-pair methods vary between 0.07 and 0.10 eV and 0.09 and 0.13 eV, respectively, with a slight overestimation of IPs being observed in comparison to CCSD.

The small error margins emphasize the accuracy and reliability of these computational methods. Most importantly, fpCC-type methods produce lower MSE and RMSE than the conventional CCSD and CCSD­(T) flavors with respect to experimental data, reducing errors by approximately a factor of more than 1.4. Similarly, good performance of the fpCC methods is observed for the experimentally known IPs of benzene and its BN-doped variants, which are reported in Table S3 of the SI.

The quality of our methods is assessed by comparing the predicted IPs with experimental results and standard electronic structure methods. Furthermore, we make predictions for the EAs, singlet–singlet, and singlet–triplet gaps exploiting the proposed DIP-EOM-fpCCSD methods (as described in section 3) and comprehensively analyze their trends and relations. Our study is further augmented with a comparative analysis of relative energies between the benzene-based derivatives and their corresponding naphthalene-based analogs. We conclude this section by predicting the most promising candidates for dye-sensitized solar cells (DSSC) and emissive devices.

#### 4.2.1 Ionization Potentials

Since vibrational effects for these systems are not reported in the literature, we performed a comparative study to assess our methods relative to both conventional approaches and experimental results. The IPs computed by both frozen-pair approaches align closely with the experimental values and CCSD­(T) results. A remarkable level of consistency is observed in the ionization potential calculations for naphthalene and its mono-BN-doped derivatives and benzene and its BN-doped variants (see Table S3 in the SI) when comparing IP-EOM-fpCCD/fpCCSD^18^ with experimental and conventional approaches such as CCSD­(T).

Based on the RMSE­(Exp.) values presented, the anticipated performance of the investigated CC models relative to the experimental results follows the order CCSD ≈ CCSD­(T) < fpCCD ≈ fpCCSD. We should note that fpCCD and fpCCSD predict IPs that are within chemical accuracy with respect to each other (about 0.04 eV or 0.92 kcal/mol).

Finally, we should stress that the cc-pVDZ basis set is sufficient for our purposes, showing differences with respect to cc-pVTZ basis sets by up to only 0.2 eV. The observed trends remain the same across frozen-pair methods and basis sets. The numerical values for selected systems are listed in Table S5 of the SI.

#### 4.2.2 Influence of BN-Doping on Molecular Properties

**Relative energies of neutral and cationic BN-doped napthalenes**: After evaluating the overall accuracy of our methodology, we now turn our attention to the trends observed in the properties of the 23 mono-BN-doped naphthalene isomers, using their relative ground state energies as a starting point. Figure 5a summarizes the neutral ground state energies in ascending order, labeled based on the relative position of their heteroatoms. Specifically, the marker shape indicates intra- or intercyclic heteroatoms (diamond and square shapes, respectively) as defined in Figure 4. Each color encodes the least amount of carbon atoms that link the boron and nitrogen atoms. This series highlights that intracyclic neutral isomers are overall more stable than their intercyclic counterparts, while systems with an even number of linking carbon atoms have lower energy than the odd ones. The last trend can be rationalized because an even number of linking carbon atoms permits neutral resonance structures without formal charges over N and B. While the odd number of linking carbons leads to zwitterionic resonance structures with a formal positive charge over the nitrogen or carbon atoms and a formal negative charge over the boron or carbon (as exemplified in Figure 4). The same trend is observed in the BN-doped benzene (azaborine) series, where the 1,2-isomer has the lowest energy, followed by the 1,4- and 1,3- ones (see Table S6 of the SI). These similarities are not only qualitative in nature but, using the partially relaxed geometry, also quantitative in character. The average ground-state energies for all intracyclic isomers with zero, one, and two linking carbon atoms (marked by the black, blue and red diamond in Figure 5, respectively) are comparable to those observed in the fully relaxed geometries of azaborines, as summarized in Table 3. That similarity suggest that each intracyclic BN-doped naphthalene isomer can be analyzed as a perturbed azaborine core.

**Figure 5:**
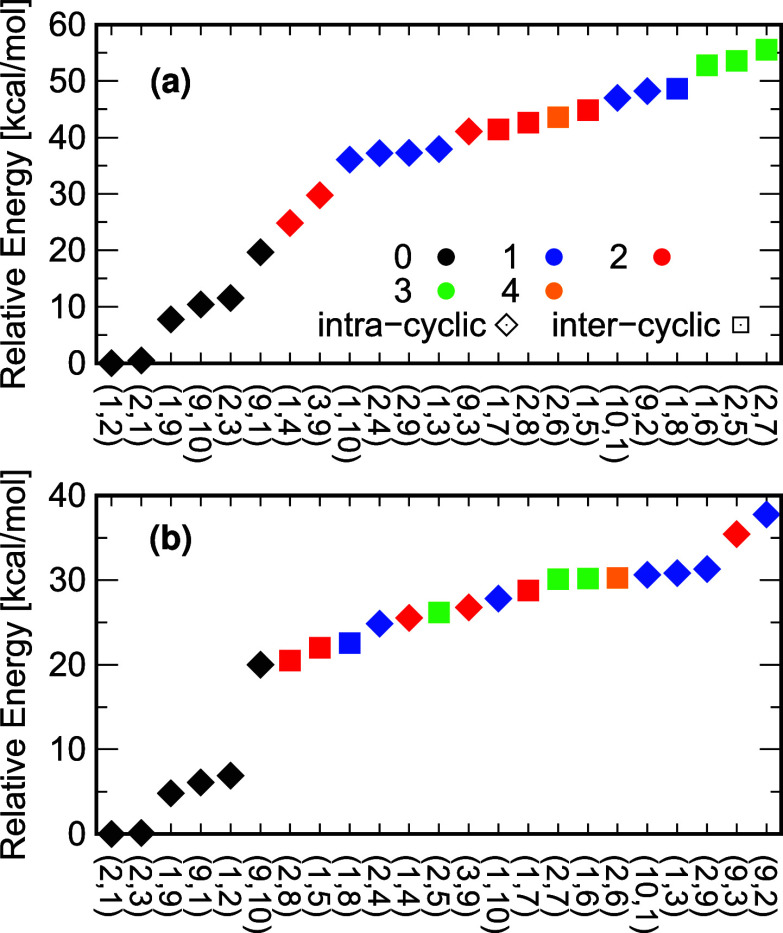
Relative energies (in kcal/mol) for the (a) neutral (fpCCSD) and (b) cationic (IP-EOM- fpCCSD) forms of the 23 mono-BN-doped naphthalene ground-state isomers for fully relaxed geometries. The colors represent the number of carbon atoms positioned between the B and N atoms in the 23 mono-BN-doped naphthalene molecules. Black indicates that B and N are adjacent, blue denotes one carbon atom between them, red corresponds to two carbon atoms, green highlights three carbon atoms, and orange shows four carbon atoms in between.

**Table 3:** Comparative Analysis of Ground-State Average Relative Energies (in kcal/mol) of Three Benzene-Based Isomers: Ortho (BN-1,2 Benzene), Meta (BN-1,3 Benzene), and Para (BN-1,4 Benzene), with Their Corresponding Neutral Naphthalene-Based Isomers Average Ground-State Energies: Ortho-Like (BN-1,2-Naphthalene), Meta-Like (BN-1,3-Naphthalene), and Para-Like (BN-1,4-Naphthalene)

	BN-naphthalene average energy						
	fully relaxed geometry			partially relaxed geometry			azaborine relative energy
BN-relative position	CCSD(T)	fpCCSD	DIP-EOM-fpCCSD^a^	CCSD(T)	fpCCSD	DIP-EOM-fpCCSD^a^	DIP-EOM-fpCCSD
(1,2)	0.0	0.0	0.0	0.0	0.0	0.0	0.0
(1,3)	29.8	32.3	28.3	36.4	38.5	35.3	30.4
(1,4)	23.7	23.6	20.9	33.5	33.4	32.2	18.9

a Excluding the BN-2,1 naphthalene total energy due to convergence issues.

In the cation case, the dependence of the ground state energy with respect to the position of the heteroatoms is not as straightforward as in its neutral counterpart (see Figure 5b). The localization of the hole governs the relative energy among the cation isomers. With few exceptions, the more localized the single-occupied orbital in a single ring, the lower the ground state total energy. For instance, all intercyclic isomers, which have the highest relative energy in the neutral ground state, are comparable with the intracyclic ones among the cations. Their highest singly-occupied molecular orbital (SOMO) resembles the neutral boratabenzene (C_5_H_6_B) and the protonated 1-benzoborinine (C_9_H_7_B) ones, that is, a well-localized hole in one ring (see Figure S2 in the SI). Similarly, among the systems with a B-N-covalent bond, the 2,3-isomer’s SOMO resembles the 1,2-azoborine one, while the 1,2 and 9-10 ones are closely related to the naphthalene SOMO. A collection of the HF natural SOMOs can be found in Figure S2 of the SI.

**IPs and EAs**: Figure 6 provides a matrix representation of IPs, EAs, singlet-singlet gaps (*E*_SS_), and singlet-triplet gaps (*E*_ST_) obtained from all 23 distinct mono-BN-doped naphthalene isomers. The cells are color-coded, with blue indicating the lowest value, whereas bronze is the highest value for each property. The positions of the nitrogen atom are displayed horizontally on top of the table, while the boron atom’s positions are listed vertically on the left. For example, BN-1,3 naphthalene (Figure 4) with nitrogen in position 1 and boron in position 3 gives values of 7.50, 0.07, 4.23, and 2.36 eV for IPs, EAs, *E*_SS_, and *E*_ST_ , respectively. This approach can be applied to all remaining isomers (see Figure 4) by mapping the positions of nitrogen and boron within each structure.

**Figure 6:**
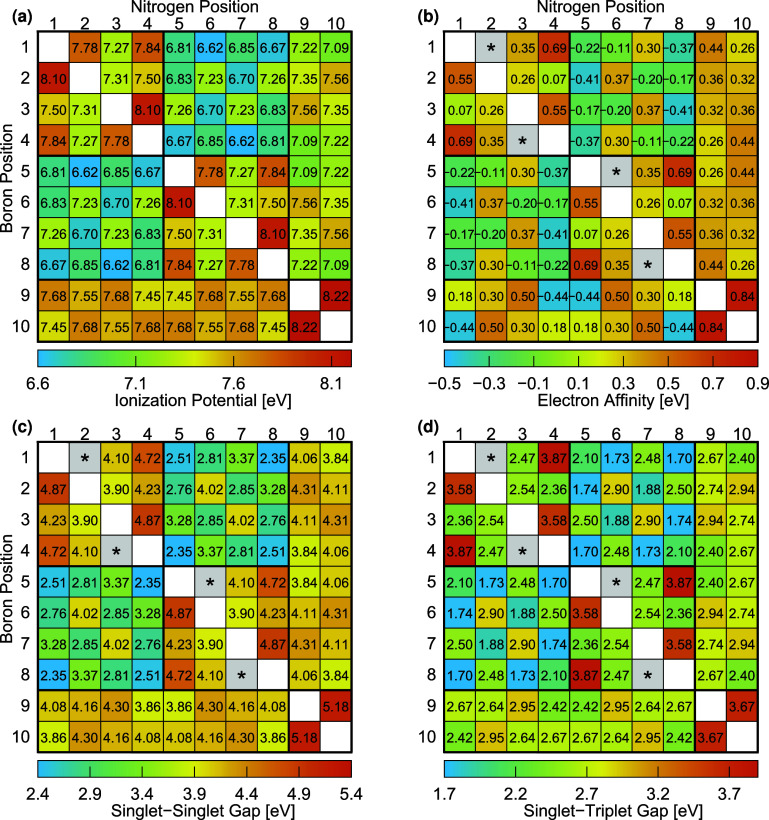
(a) Ionization potential (determined by IP-EOM-fpCCSD), (b) electron affinity (calculated using eq 11), (c) singlet–singlet gap, and (d) singlet–triplet gap (both computed using eq 12 based on DIP-EOM-fpCCSD) are measured in electron volts (eV) for 23 mono-BN-doped naphthalene isomers using fully relaxed geometries. A star (★) indicates systems that encountered convergence issues with the dianion reference state.

Extending our discussion on the distinct trends observed for the relative energies of the neutral and cation ground states in dependence on the heteroatom’s relative positions, we can rationalize the observed trends in IPs and EAs. The intercyclic isomers increase the energy of the neutral ground state while simultaneously reducing it for the cation state, resulting in substantially smaller intercyclic IPs compared to intracyclic ones (see the two bluish off-diagonal blocks in Figure 6a). However, some intracyclic isomers also exhibit smaller IP values, which are present in diagonal blocks of Figure 6. This behavior is primarily influenced by the presence of an odd number of carbon atoms between heteroatoms (B and N), as observed in BN-10,1 naphthalene (IP = 7.09 eV). In contrast, an even number of carbon atoms shows comparatively higher IPs. A similar trend of low values in other spectral properties is also observed in electron affinity, the singlet–singlet gap, and the singlet–triplet gap, as seen in BN-1,10 naphthalene (EA = −0.44 eV), BN-1,10 naphthalene (*E*_SS_ gap = 3.86 eV) and BN-10,1 naphthalene ( *E*_SS_ gap = 3.84 eV), and BN-1,10 naphthalene (*E*_ST_ gap = 2.42 eV) and BN-10,1 naphthalene (*E*_ST_ gap = 2.40 eV), respectively. A second noticeable pattern is observed, showing relatively lower IPs for the C-N bridged isomers (BN-9,1-naphthalene, BN-9,2-naphthalene, BN-9,3-naphthalene, and BN-10,1-naphthalene in the 9th and 10th columns) compared to the C-B bridged isomers (BN-1,9-naphthalene, BN-2,9-naphthalene, BN-3,9-naphthalene, and BN-1,10-naphthalene in the 9th and 10th rows).

The EAs follow a pattern largely consistent with that of the IPs. The off-diagonal block of the intercyclic isomers has overall lower values. From an orbital perspective, the anionic ground states exhibit lower energy as the singly occupied molecular orbital (SOMO) becomes more localized over the nitrogen-containing ring. This shift in the localization of the openshell orbital from boron to nitrogen also swaps the relative energies between C-B and C-N bridged isomers. In contrast, the former has lower relative energies for the anion.

**Singlet–Singlet and Singlet–Triplet Gaps**: The lowest-lying singlet and triplet excited states of all mono-BN-doped naphthalene isomers are dominated by HOMO-to-LUMO transitions. These orbitals closely resemble the cation and anion SOMO, respectively (see Figure S3 in the SI). Consequently, similar intra- and intercyclic patterns are observed in both the singlet–singlet (S_1_–S_0_) and singlet–triplet (T_1_–S_0_) energy gaps, with no significant energy difference between C-B and C-N bridged isomers. Based on these results, mono-BN-doped naphthalene isomers exhibit their potential for fine-tuning color emissions across the visible spectrum. We observed the five lowest singlet–singlet gaps between 2.35 and 2.85 eV, which emerge from the intercyclic heteroatom arrangements in BN-1,5-naphthalene (2.51 eV), BN-1,6-naphthalene (2.76 eV), BN-1,8-naphthalene (2.35 eV), BN-2,5-naphthalene (2.81 eV), and BN-2,7-naphthalene (2.85 eV). These gaps enable absorption and emission in the visible range, making these isomers ideal for displaying colors from orange to purple in emissive devices. Moreover, the charge splitting caused by the localization of singly occupied orbitals in the excited states of intercyclic isomers makes them highly suitable as efficient dye-sensitizers for solar cells. The highest S_1_–S_0_ gaps are observed in the intracyclic heteroatom arrangements of BN-1,2-naphthalene (4.87 eV), BN-1,3-naphthalene (4.23 eV), and BN-1,4-naphthalene (4.72 eV) (see Figure 6), indicating strong UV absorption, making them particularly suitable for integration into multilayered lighting devices to boost performance. In contrast, the five compounds with the lowest singlet–triplet gaps, ranging from 1.70 to 2.10 eV, are observed in BN-1,5 (2.10 eV), BN-1,6 (1.74 eV), BN-1,8 (1.70 eV), BN-2,5 (1.73 eV), and BN-2,7 (1.88 eV) (see Figure 6). These gaps suggest potential phosphorescence emission, which could increase the duration of emission and ensure stable OLED colors. This tunability enables precise control of color emission across the full spectrum, from red to blue, making BN-doped naphthalene versatile materials for high-quality display applications.

## 5 Conclusions and Outlook

In this work, we derived the working equations for the DIP-EOM-fpCCD, DIP-EOM-fpCCSD, DIP-EOM-fpLCCD, and DIP-EOM-fpLCCSD methods and implemented them in the PyBEST software package.^74,75^ These methods are based on the pCCD reference wave function, which can use both canonical HF or pCCD natural orbitals (after orbital optimization).

The newly developed DIP-EOM-fpCC models were carefully tested on the singlet–triplet gaps of ortho-, meta-, and para-benzynes, for which experimental and theoretical reference data are available. The frozen-pair (fp)­CC formalism and its linearized (L)­CC version incorporate dynamical correlation, which significantly improved their performance compared to our previous DIP-EOM-pCCD method in the study of benzyne isomers.^92^ These approaches achieve accuracy comparable to standard DIP-EOM-CCSD and CCSD­(T), but at a lower computational cost than CCSD­(T). DIP-EOM-fpCCD and DIP-EOM-fpCCSD are the most consistent across the benzyne series and investigated basis sets. To that end, these models were later used to explore the spectral properties of the mono-BN-doped napthalene isomers.

Our statistical analysis, based on mean signed and root mean square errors, demonstrates that the frozen-pair coupled cluster formalisms (IP-EOM-fpCCD and IP-EOM-fpCCSD) show higher accuracy over conventional methods (CCSD and CCSD­(T)) when compared to experimental data. However, these approaches exhibit a slight overestimation of IPs with respect to CCSD­(T). The computed mean signed error is −0.15 eV for IP-EOM-fpCCD and −0.19 eV for IP-EOM-fpCCSD, and the root mean square error is 0.20 eV for the former and 0.21 eV for the latter relative to the experimental data, while their deviations from the standard CCSD­(T) method are 0.10 eV and 0.07 eV, respectively. These results ensure that the proposed methods are accurate and reliable for exploring the electronic structure and predicting other properties of mono-BN-doped naphthalene isomers.

This study further explores how the spatial arrangement of nitrogen and boron atoms affects the electronic properties of mono-BN-doped naphthalene isomers, with the objective of utilizing these systems as core structural units to design novel extended molecules.^106^ The main observed structural feature that affects spectral properties, such as IPs, EAs, and singlet–singlet and singlet–triplet gaps, is the intra- or intercyclic character of the isomer. First, heteroatoms within a single ring exhibit larger spectral values than those distributed across two different rings. The second structural factor is the number of carbon atoms that link the heteroatoms. An even number of carbon atoms generally produces higher spectral values than an odd number. In comparison, fewer linking carbons show a slight correlation with higher values. The relative energies of intracyclic isomers are lower than those of intercyclic, which makes them more stable. These relative energies correlate with higher IPs, EAs, singlet–singlet, and singlet–triplet gap values. Moreover, C–B and C–N bridged isomers yield intermediate spectral values, whereas the B–N bridged isomer (BN-9,10-naphthalene) ranks among the highest. The overall lower energy gaps of the intercyclic isomer display a significant charge transfer in the excited state and facilitate electronic transitions in the visible region of the spectra of mono-BN-doped naphthalenes. These properties are desirable for any system with potential applications as dye-sensitized solar cells.

Finally, our work also demonstrates that the electronic properties of BN-doped naphthalene follow a trend similar to the ones observed for BN-doped benzene. To that end, results for the naphthalene series can be used as a starting point for innovative quantum chemistry-driven molecular designs of larger polycyclic aromatic hydrocarbons. The affordable computational scaling (up to $O(N^{6})$ ) and confirmed predictive power of the DIP-EOM-fpCCD and DIP-EOM-fpCCSD methods open the way for reliable modeling of large organic molecules, including BN-doped benzene and naphthalene isomers. Observing patterns in this work will contribute to establishing a robust foundation for future advancements and provide crucial guidance to chemical scientists in synthesizing and customizing organic electronic materials tailored toward specific properties.

## Supplementary Material



## Data Availability

The data underlying this study are available in the published article and its Supporting Information. The released version of the PyBEST code is available on Zenodo at https://zenodo.org/records/10069179 and on PyPI at https://pypi.org/project/pybest/.

## Notes

Working equations of DIP-EOM-fpCC, geometries of naphthalene, benzene and its BN-doped variants, cc-pVTZ data, vertical excitation energies for benzynes, selected SOMO orbitals, relative energy comparison of BN-naphthalene vs BN-benzene (PDF)
