# Supplementary material for: Frozen-Pair-Type pCCD-Based Methods and Their Double Ionization Variants to Predict Properties of Prototypical BN-Doped Light Emitters
Source: J Chem Theory Comput. 2025 May 14;21(10):5049–61. doi: 10.1021/acs.jctc.5c00057 (PMC12120922; doi:10.1021/acs.jctc.5c00057)
Supplement: Supplementary file 1 [file ct5c00057_si_001.pdf]

# Frozen-Pair-Type pCCD-Based Methods and their Double Ionization Variants to Predict Properties of Prototypical BN-Doped Light Emitters

Ram Dhari Pandey,[a] Matheus Morato F. de Moraes,[b*] Katharina Boguslawski,[a*] and Paweł Tecmer[a*]

[a] *Institute of Physics, Faculty of Physics, Astronomy and Informatics, Nicolaus Copernicus University in Toruń, Grudziądzka 5, 87-100 Toruń, Poland*

[b] *Department of Chemistry, University of Louisville, 2320 S. Brook St. Louisville, Kentucky 40292, United States*

*Email: matheusmorat@gmail.com, k.boguslawski@fizyka.umk.pl, ptecmer@fizyka.umk.pl

## Supplementary Information

# S1 DIP-EOM-fpCC and DIP-EOM-fpLCC working equations

For $S_z = 0$ states, the operator $\hat{R}^{\mathrm{DIP}}(k)$ is restricted to the following spin blocks

$$\begin{aligned}\hat{R}^{S_z=0}(k) &= \sum_{i\bar{j}} r_{i\bar{j}}(k)\hat{\bar{j}}\hat{i} + \frac{1}{2}\sum_{i\bar{j}k}\sum_{a} r_{i\bar{j}k}{}^{a}(k)\hat{a}^{\dagger}\hat{k}\hat{\bar{j}}\hat{i} + \frac{1}{2}\sum_{i\bar{j}\bar{k}}\sum_{\bar{a}} r_{i\bar{j}\bar{k}}{}^{\bar{a}}(k)\hat{\bar{a}}^{\dagger}\hat{\bar{k}}\hat{\bar{j}}\hat{i} \\ &= \hat{R}^{\beta\alpha}_{\mathrm{2h}}(k) + \hat{R}^{\alpha\alpha\beta\alpha}_{\mathrm{3h1p}}(k) + \hat{R}^{\beta\beta\beta\alpha}_{\mathrm{3h1p}}(k), \end{aligned} \tag{1}$$

while the configurational subspace during diagonalization is spanned by $\left|\Phi_{i\bar{j}}\right\rangle, \left|\Phi_{i\bar{j}k}{}^{a}\right\rangle, \left|\Phi_{i\bar{j}\bar{k}}{}^{\bar{a}}\right\rangle$. The working equations for the various DIP-EOM flavors mentioned here read as follows (for each $\hat{R}$ block)

$$\begin{aligned}(\bar{H}\hat{R})_{i\bar{j}} &= I_{jm}R_{i\bar{m}} + I_{im}R_{m\bar{j}} + I_{iJlM}R_{l\bar{m}} + I_{kc}R_{i\bar{j}m}{}^{d} + I_{kc}R_{i\bar{j}\bar{m}}{}^{\bar{d}} \\ &\quad + I_{jmKC}R_{i\bar{m}k}{}^{c} + I_{jmkc}R_{i\bar{m}\bar{k}}{}^{\bar{c}} + I_{imkc}R_{m\bar{j}k}{}^{c} + I_{imKC}R_{m\bar{j}\bar{k}}{}^{\bar{c}}\end{aligned} \tag{2}$$

$$\begin{aligned}(\bar{H}\hat{R})_{i\bar{j}k}{}^{c} &= I_{JkcM}R_{i\bar{m}} + I_{ikcm}R_{m\bar{j}} + P(i/k)I_{iJkclm}R_{l\bar{m}} + P(i/k)I_{km}R_{i\bar{j}m}{}^{c} + \frac{1}{2}P(i/k)I_{jm}R_{i\bar{m}k}{}^{c} \\ &+ \frac{1}{2}P(i/k)I_{cd}R_{i\bar{j}k}{}^{d} + P(i/k)I_{kcMD}R_{i\bar{j}m}{}^{d} + P(i/k)I_{kCmD}R_{i\bar{j}m}{}^{d} \\ &+ \frac{1}{2}P(i/k)I_{jCmD}R_{i\bar{m}k}{}^{d} + P(i/k)I_{kcMD}R_{i\bar{j}\bar{m}}{}^{\bar{d}} \\ &+ P(i/k)I_{iJlM}R_{l\bar{m}k}{}^{c} + P(i/k)I_{iklm}R_{l\bar{j}m}{}^{c} \\ &- \frac{1}{2}P(i/k)\left(\langle ml||ed\rangle\, t^{ce}_{kj}\right)R_{i\bar{m}\bar{l}}{}^{\bar{d}} - P(i/k)\left(\langle ml|ed\rangle\, t^{ce}_{kj}\right)R_{i\bar{m}l}{}^{d} \\ &- \frac{1}{4}P(i/k)\left(\langle ml||ed\rangle\left(t^{ec}_{ik} - t^{ce}_{ik}\right)\right)R_{m\bar{j}l}{}^{d} - \frac{1}{2}P(i/k)\left(\langle ml|ed\rangle\left(t^{ec}_{ik} - t^{ce}_{ik}\right)\right)R_{m\bar{j}\bar{l}}{}^{\bar{d}}\end{aligned} \tag{3}$$

$$\begin{aligned}(\bar{H}\hat{R})_{i\bar{j}\bar{k}}{}^{\bar{c}} &= I_{jkcm}R_{i\bar{m}} + I_{IkcM}R_{m\bar{j}} + P(j/k)I_{Jikclm}R_{l\bar{m}} + P(j/k)I_{km}R_{i\bar{j}\bar{m}}{}^{\bar{c}} + \frac{1}{2}P(j/k)I_{im}R_{m\bar{j}\bar{k}}{}^{\bar{c}} \\ &+ \frac{1}{2}P(j/k)I_{cd}R_{i\bar{j}\bar{k}}{}^{\bar{d}} + P(j/k)I_{kcMD}R_{i\bar{j}\bar{m}}{}^{\bar{d}} + P(j/k)I_{kCmD}R_{i\bar{j}\bar{m}}{}^{\bar{d}} \\ &+ \frac{1}{2}P(j/k)I_{iCmD}R_{m\bar{j}\bar{k}}{}^{\bar{d}} + P(j/k)I_{kcMD}R_{i\bar{j}m}{}^{d} \\ &+ P(j/k)I_{iJlM}R_{l\bar{m}\bar{k}}{}^{\bar{c}} + P(j/k)I_{jklm}R_{i\bar{l}\bar{m}}{}^{\bar{c}} \\ &- \frac{1}{2}P(j/k)\left(\langle ml|ed\rangle\left(t^{ec}_{jk} - t^{ce}_{jk}\right)\right)R_{i\bar{m}l}{}^{d} - \frac{1}{4}P(j/k)\left(\langle ml||ed\rangle\, t^{ce}_{kj}\right)R_{i\bar{m}\bar{l}}{}^{\bar{d}} \\ &- \frac{1}{2}P(j/k)\left(\langle ml||ed\rangle\, t^{ec}_{ik}\right)R_{m\bar{j}l}{}^{d} - P(j/k)\left(\langle ml|ed\rangle\, t^{ec}_{ik}\right)R_{m\bar{j}\bar{l}}{}^{\bar{d}}\end{aligned} \tag{4}$$

with spin-free amplitudes ($t_{ij}^{ab} = t_{i\bar{j}}^{a\bar{b}}$) and intermediates (all integrals are represented in the restricted orbital basis, that is, $\langle pq|rs\rangle = \langle p\bar{q}|r\bar{s}\rangle = \langle \bar{p}q|\bar{r}s\rangle = \langle \bar{p}\bar{q}|\bar{r}\bar{s}\rangle$; summation over repeated indices is implied)

$$\begin{aligned} I_{im}(i,m) = &-f_{im} - f_{md}t_i^d - \left(\langle ml||id\rangle + \langle ml|id\rangle\right) t_l^d - \frac{1}{2}\langle ml||ed\rangle \left(t_{il}^{ed} - t_{il}^{de}\right) \\ &- \left(\langle ml||ed\rangle + \langle ml|ed\rangle\right) t_i^e t_l^d - \langle ml|ed\rangle\, t_{il}^{ed} \end{aligned} \quad (5)$$

$$I_{jm} = I_{im}(j,m)$$

$$I_{km} = I_{im}(k,m)$$

$$I_{iJkclm}(i,j,k,c,l,m) = \langle lm|id\rangle\, t_{jk}^{dc} + \langle lm|ed\rangle\, t_i^e t_{jk}^{dc} + \frac{1}{2}\langle lm|dj\rangle \left(t_{ik}^{dc} - t_{ik}^{cd}\right) + \frac{1}{2}\langle lm|de\rangle\, t_j^e (t_{ik}^{dc} - t_{ik}^{cd}) \quad (6)$$

$$I_{Jikclm} = I_{iJkclm}(j,i,k,c,m,l)$$

$$I_{kc} = f_{kc} + \left(\langle mk||dc\rangle + \langle mk|dc\rangle\right) t_m^d \quad (7)$$

$$I_{iJlM} = \langle ij|lm\rangle + \langle lm|id\rangle\, t_j^d + \langle lm|dj\rangle\, t_i^d + \langle lm|cd\rangle\, t_{ij}^{cd} + \langle lm|cd\rangle\, t_i^c t_j^d \quad (8)$$

$$I_{iklm}(i,k,l,m) = \frac{1}{4}\langle ik||lm\rangle + \frac{1}{2}\langle lm||ek\rangle\, t_i^e + \frac{1}{8}\langle lm||ed\rangle \left(t_{ik}^{ed} - t_{ik}^{de}\right) + \frac{1}{4}\langle lm||ed\rangle\, t_i^e t_k^d \quad (9)$$

$$I_{jklm} = I_{iklm}(j,k,l,m)$$

$$I_{imKC}(i,m,k,c) = -\langle ic|mk\rangle - \langle mk|dc\rangle\, t_i^d \quad (10)$$

$$I_{jmKC} = I_{imKC}(j,m,k,c)$$

$$I_{imkc}(i,m,k,c) = -\frac{1}{2}\langle ic||mk\rangle - \frac{1}{2}\langle mk||dc\rangle\, t_i^d \tag{11}$$

$$I_{jmkc} = I_{imkc}(j,m,k,c)$$

$$\begin{aligned} I_{JkcM}(j,k,c,m) = &-\langle mc|jk\rangle - f_{md}t_{jk}^{dc} + \langle ml|jk\rangle\, t_l^c - \langle mc|jd\rangle\, t_k^d - \langle mc|dk\rangle\, t_j^d \\ &+ \langle ml|jd\rangle\, t_k^d t_l^c + \langle ml|dk\rangle\, t_j^d t_l^c - \langle mc|de\rangle\, t_k^e t_j^d \\ &- \langle ml|jd\rangle\,(t_{kl}^{cd} - t_{lk}^{cd}) - \langle ml||jd\rangle\, t_{kl}^{cd} + \langle ml|dk\rangle\, t_{jl}^{dc} - \langle mc|de\rangle\, t_{jk}^{de} \\ &- \langle ml|ed\rangle\, t_l^d t_{kj}^{ce} - \langle ml||ed\rangle\, t_l^d t_{kj}^{ce} - \langle ml|ed\rangle\, t_j^e(t_{lk}^{dc} - t_{kl}^{dc}) - \langle ml||ed\rangle\, t_j^e t_{lk}^{dc} \\ &+ \langle ml|ed\rangle\, t_k^d t_{lj}^{ce} + \langle ml|ed\rangle\, t_l^c t_{kj}^{de} + \langle ml|ed\rangle\, t_l^c t_k^d t_j^e \end{aligned} \tag{12}$$

$$I_{IkcM} = I_{JkcM}(i,k,c,m)$$

$$\begin{aligned} I_{ikcm}(i,k,c,m) = &-\frac{1}{2}\langle mc||ik\rangle - f_{md}(t_{ki}^{cd} - t_{ki}^{dc}) + \frac{1}{2}\langle ml||ik\rangle\, t_l^c - \langle mc||id\rangle\, t_k^d \\ &+ \langle ml||id\rangle\, t_k^d t_l^c - \frac{1}{2}\langle mc||de\rangle\, t_k^e t_i^d \\ &- \langle ml||id\rangle\,(t_{kl}^{cd} - t_{lk}^{cd}) - \langle ml|id\rangle\, t_{kl}^{cd} - \frac{1}{4}\langle mc||de\rangle\,(t_{ik}^{de} - t_{ki}^{de}) \\ &- \frac{1}{2}\langle ml||ed\rangle\, t_l^d(t_{ki}^{ce} - t_{ik}^{ce}) - \frac{1}{2}\langle ml|ed\rangle\, t_l^d(t_{ki}^{ce} - t_{ik}^{ce}) - \langle ml||ed\rangle\, t_i^e(t_{lk}^{dc} - t_{kl}^{dc}) \\ &- \langle ml|ed\rangle\, t_i^e t_{lk}^{dc} + \frac{1}{4}\langle ml||ed\rangle\, t_l^c(t_{ki}^{de} - t_{ik}^{de}) + \frac{1}{2}\langle ml||ed\rangle\, t_l^c t_k^d t_i^e \end{aligned} \tag{13}$$

$$I_{jkcm} = I_{ikcm}(j,k,c,m)$$

$$\begin{aligned} I_{cd} = &f_{cd} - f_{ld}t_l^c + (\langle cl||de\rangle + \langle cl|de\rangle)\, t_l^e - \frac{1}{2}\langle lm||de\rangle\,(t_{lm}^{ce} - t_{ml}^{ce}) \\ &- (\langle lm||de\rangle + \langle lm|de\rangle)\, t_l^c t_m^e - \langle lm|de\rangle\, t_{lm}^{ce} \end{aligned} \tag{14}$$

$$I_{iCmD}(i,c,m,d) = -\langle cm|di\rangle + \langle ml|id\rangle\, t_l^c - \langle cm|de\rangle\, t_i^e + \langle lm|de\rangle\, t_i^e t_l^c + \langle lm|de\rangle\, t_{li}^{ce} \tag{15}$$

$$I_{jCmD} = I_{iCmD}(j,c,m,d)$$

$$I_{kCmD} = I_{iCmD}(k,c,m,d)$$

$$I_{kcMD} = \langle cm|kd\rangle - \langle ml|dk\rangle\, t_l^c + \langle cm|ed\rangle\, t_k^e - \langle lm|ed\rangle\, t_k^e t_l^c + \langle lm|ed\rangle\, (t_{lk}^{ec} - t_{kl}^{ec}) + \langle lm||ed\rangle\, t_{lk}^{ec} \quad (16)$$

For the linearized version of fpCCSD, we approximate the disconnected $\hat{T}_1\hat{T}_2$ term with $\hat{T}_1\hat{T}_\mathrm{p}$, while all remaining disconnected terms are neglected. We also store both pair and broken-pair amplitudes in the $\hat{T}_2$ tensor, that is, $\hat{T}_2 = \hat{T}_2' + \hat{T}_\mathrm{p}$. Thus, we obtain the following modified set of intermediates (using the spin-free amplitudes and integrals represented in the spatial orbital basis)

$$I_{im}^{\mathrm{fpLCC}} = -f_{im} - f_{md} t_i^d - (\langle ml||id\rangle + \langle ml|id\rangle)\, t_l^d - \frac{1}{2}\langle ml||ed\rangle \left(t_{il}^{ed} - t_{il}^{de}\right) - \langle ml|ed\rangle\, t_{il}^{ed} \quad (17)$$

$$I_{jm}^{\mathrm{fpLCC}} = I_{im}^{\mathrm{fpLCC}}(j,m) \quad (18)$$

$$I_{kc}^{\mathrm{fpLCC}} = I_{kc} \quad (19)$$

$$I_{iJlM}^{\mathrm{fpLCC}} = \langle ij|lm\rangle + \langle lm|id\rangle\, t_j^d + \langle lm|dj\rangle\, t_i^d + \langle lm|cd\rangle\, t_{ij}^{cd} \quad (20)$$

$$I_{iklm}^{\mathrm{fpLCC}} = \frac{1}{4}\langle ik||lm\rangle + \frac{1}{2}\langle lm||ek\rangle\, t_i^e + \frac{1}{8}\langle lm||ed\rangle \left(t_{ik}^{ed} - t_{ik}^{de}\right) \quad (21)$$

$$I_{jklm}^{\mathrm{fpLCC}} = I_{iklm}^{\mathrm{fpLCC}}(j,k,l,m) \quad (22)$$

$$I_{imKC}^{\mathrm{fpLCC}} = I_{imKC} \quad (23)$$

$$I_{imkc}^{\mathrm{fpLCC}} = I_{imkc} \quad (24)$$

$$\begin{aligned} I_{JkcM}^{\mathrm{fpLCC}} = &- \langle mc|jk\rangle - f_{md} t_{jk}^{dc} + \langle ml|jk\rangle\, t_l^c - \langle mc|jd\rangle\, t_k^d - \langle mc|dk\rangle\, t_j^d \\ &- \langle ml|jd\rangle\, (t_{kl}^{cd} - t_{lk}^{cd}) - \langle ml||jd\rangle\, t_{kl}^{cd} + \langle ml|dk\rangle\, t_{jl}^{dc} - \langle mc|de\rangle\, t_{jk}^{de} \\ &+ \left[- \langle ml|cd\rangle\, t_l^d t_{jj}^{cc} - \langle ml||cd\rangle\, t_l^d t_{jj}^{cc} + \langle ml|dd\rangle\, t_l^c t_{jj}^{dd}\right] \delta_{jk} \\ &- \langle mk||ec\rangle\, t_j^e t_{kk}^{cc} + \langle mj|cd\rangle\, t_k^d t_{jj}^{cc} \end{aligned} \quad (25)$$

$$
\begin{aligned}
I^{\text{fpLCC}}_{ikcm} = &-\frac{1}{2}\langle mc||ik\rangle - f_{md}(t^{cd}_{ki} - t^{dc}_{ki}) + \frac{1}{2}\langle ml||ik\rangle\, t^{c}_{l} - \langle mc||id\rangle\, t^{d}_{k} \\
&- \langle ml||id\rangle\,(t^{cd}_{kl} - t^{cd}_{lk}) - \langle ml|id\rangle\, t^{cd}_{kl} - \frac{1}{4}\langle mc||de\rangle\,(t^{de}_{ik} - t^{de}_{ki}) \\
&- \langle mk|ec\rangle\, t^{e}_{i} t^{cc}_{kk}
\end{aligned}
\tag{26}
$$

$$
I^{\text{fpLCC}}_{cd} = f_{cd} - f_{ld}t^{c}_{l} + (\langle cl||de\rangle + \langle cl|de\rangle)\, t^{e}_{l} - \frac{1}{2}\langle lm||de\rangle\,(t^{ce}_{lm} - t^{ce}_{ml}) - \langle lm|de\rangle\, t^{ce}_{lm} \tag{27}
$$

$$
I^{\text{fpLCC}}_{iCmD} = -\langle cm|di\rangle + \langle ml|id\rangle\, t^{c}_{l} - \langle cm|de\rangle\, t^{e}_{i} + \langle lm|de\rangle\, t^{ce}_{li} \tag{28}
$$

$$
I^{\text{fpLCC}}_{jCmD} = I^{\text{fpLCC}}_{iCmD}(j, c, m, d)
$$

$$
I^{\text{fpLCC}}_{kCmD} = I^{\text{fpLCC}}_{iCmD}(k, c, m, d)
$$

$$
I^{\text{fpLCC}}_{kcMD} = \langle cm|kd\rangle - \langle ml|dk\rangle\, t^{c}_{l} + \langle cm|ed\rangle\, t^{e}_{k} + \langle lm|ed\rangle\,(t^{ec}_{lk} - t^{ec}_{kl}) + \langle lm||ed\rangle\, t^{ec}_{lk} \tag{29}
$$

To arrive at the intermediates of the fpCCD or fpLCCD flavors, all $\hat{T}_1$ terms have to be deleted in the above equations. Since $\hat{T}_2$ is solely coupled to $\hat{T}_1$, the intermediates (effective Hamiltonian terms) of fpCCD and fpLCCD are formally identical and the corresponding DIP-EOM formalisms only differ in the values of the $\hat{T}_2$ amplitudes.

# S2 Benzyne isomers: Comparison with other methods in the cc-pVTZ basis set

| Methods | ortho-benzyne | | meta-benzyne | | para-benzyne | |
|---|---|---|---|---|---|---|
| | $^1A_1$ | $^3B_2$ | $^1A_1$ | $^3B_2$ | $^1A_g$ | $^3B_{1u}$ |
| DIP-EOM-pCCD | -229.85076 | 1.519 | -229.83641 | 0.950 | -229.81474 | 0.325 |
| DIP-EOM-fpCCD | -230.37797 | 1.762 | -230.35630 | 0.764 | -230.34705 | 0.339 |
| DIP-EOM-fpLCCD | -230.39424 | 1.775 | -230.37233 | 0.746 | -230.36416 | 0.330 |
| DIP-EOM-fpCCSD | -230.39839 | 1.783 | -230.37878 | 0.837 | -230.36512 | 0.309 |
| DIP-EOM-fpLCCSD | – | – | -230.40595 | 0.858 | -230.39336 | 0.177 |
| DIP-EOM-CCSD[c] | – | – | – | – | -230.35601 | 0.360 |
| DIP-EOM-CCSD[a, 1] | | 1.847 | | 0.854 | | 0.191 |
| DEA-EOM-CCSD[2] | | 1.625 | | 0.799 | | 0.145 |
| EOM-pCCD-TCCSD[a, 3] | – | 1.531 | – | 0.785 | – | −0.09 |
| EOM-pCCD-TCCSD[b, 3] | – | 1.526 | – | 0.828 | – | 0.130 |
| CCSD[4] | -230.416059 | 1.331 | -230.391192 | 0.572 | -230.347495 | −0.746 |
| CCSD[a, 1] | | 1.327 | | 0.455 | | −0.833 |
| CCSD(T)[a, 1] | | 1.604 | | 0.958 | | 0.156 |
| SF-CCSD[5] | -230.42486 | 1.578 | -230.40162 | 0.782 | -230.38010 | 0.147 |
| SF-CCSD(fT)[5] | | 1.615 | | 0.875 | | 0.169 |
| SF-CIS[6] | -229.49504 | 1.007 | -229.47187 | 0.166 | -229.46472 | 0.014 |
| SF-CIS(D)[6] | -230.45684 | 1.548 | -230.38757 | 0.842 | -230.41234 | 0.092 |
| SF-OD[6] | -230.50269 | 1.632 | -230.47817 | 0.837 | -230.45743 | 0.171 |
| Experimental[7,8] | | 1.628±0.013 | | 0.911±0.014 | | 0.165±0.016 |
| ΔZPE[9] | | −0.028 | | 0.043 | | 0.021 |
| Experimental-ΔZPE | | 1.656 | | 0.868 | | 0.144 |

[a] calculations performed in canonical HF orbitals and a different geometry than used in this study
[b] calculations performed in response pCCD orbitals and a different geometry than used in this study. These orbitals do not correspond to pCCD-optimized orbitals.
[c] calculations performed in canonical HF orbitals and the same geometry as used in this study
⋆ CCSD and CCSD(T) refer to energy differences for state-specific calculations (singlet and triplet state) using an older cc-pVDZ basis set and slightly different geometries (see Ref. 4)
† not reported due to convergence difficulties of the pCCD-fpLCCSD dianion reference state for the singlet state; the state converged, but the energy is suspiciously high.

Table S1: Total ground-state energies in $E_h$ and adiabatic excitation energies to the lowest-lying triplet states of ortho-, meta-, and para-benzyne were computed in electron volts (eV) with the cc-pVTZ basis set.

Table S1 summarizes the singlet-triplet gaps in all benzyne isomers determined for various methods and the cc-pVTZ basis set. We should stress that different geometries have been used (as indicated in the Table). In general, the performance of EOM-pCCD-TCCSD should be similar to DIP-EOM-fpCCSD as both represent the same DIP-EOM model. They employ different molecular orbitals (relaxed pCCD natural orbitals vs. pCCD-optimized natural orbitals) and molecular structures. The better agreement of EOM-pCCD-TCCSD with experimental data might originate from the chosen molecular geometry. A similar observation can be made for the different DIP-EOM-CCSD singlet-triplet gaps, which have been determined for different molecular structures. We should stress that the DZ-quality optimized molecular structures used in this work result in higher-lying adiabatic singlet-triplet gaps compared to vertical ones (see also Table S2 below) for para-benzyne.

This effect is particularly pronounced for para-benzyne and is smallest for meta-benzyne, where both approaches give similar singlet-triplet gaps. Furthermore, our DIP-EOM-fpCCSD/DZ results for para-benzyne (0.131 eV) agree well with the EOM-pCCD-TCCSD/TZ results (0.130 eV). Finally, the EOM-pCCD-TCCSD/TZ and the DIP-fpCCSD/TZ results for para-benzyne are closer to experimental results than the conventional DIP-EOM-CCSD ones. This observation suggests that decoupling the seniority-zero sector from the remaining sectors improves the performance of DIP-EOM-CC when static correlation becomes significant.

# S3 Benzyne isomers: Vertical excitation energies

| Methods | ortho-benzyne | | meta-benzyne | | para-benzyne | |
|---|---|---|---|---|---|---|
| | $^1A_1$ | $^3B_2$ | $^1A_1$ | $^3B_2$ | $^1A_g$ | $^3B_{1u}$ |
| cc-pVDZ | | | | | | |
| DIP-EOM-fpCCD | -230.16554 | 2.560 | -230.14968 | 1.743 | -230.14249 | 0.245 |
| DIP-EOM-fpLCCD | -230.19094 | 2.546 | -230.17535 | 1.736 | -230.16976 | 0.245 |
| DIP-EOM-fpCCSD | -230.16554 | 2.621 | -230.16784 | 1.776 | -230.15690 | 0.260 |
| DIP-EOM-fpLCCSD | -230.22142 | 2.725 | -230.20199 | 1.769 | -230.19280 | 0.265 |
| cc-pVTZ | | | | | | |
| DIP-EOM-fpCCD | -230.37797 | 2.665 | -230.35630 | 0.764 | -230.34705 | 0.242 |
| DIP-EOM-fpLCCD | -230.39424 | 2.656 | -230.37233 | 0.746 | -230.36416 | 0.241 |
| DIP-EOM-fpCCSD | -230.39839 | 2.753 | -230.37878 | 0.837 | -230.36512 | 0.255 |
| DIP-EOM-fpLCCSD | † | † | -230.40595 | 0.858 | -230.39336 | 0.255 |

Table S2: Total ground-state energies in hartree and vertical excitation energies to the lowest-lying triplet states of ortho-, meta-, and para-benzyne were computed in electron volts (eV) with the cc-pVDZ and cc-pVTZ basis sets. † Not reported due to convergence issues.

# S4 Benzene and its BN-doped variants

| Molecule | Fully relaxed geometry | | | | Partially relaxed geometry | | | | | Exp. |
|---|---|---|---|---|---|---|---|---|---|---|
| | standard methods | | IP-EOM- | | standard methods | | | IP-EOM- | | |
| | CCSD | CCSD(T) | fpCCD | fpCCSD | CCSD | CCSD(T) | pCCD | fpCCD | fpCCSD | |
| benzene | 9.10 | 9.13 | 9.20 | 9.16 | 9.28 | 9.35 | 7.15 | 9.18 | 9.14 | 9.23[10] |
| 1,2-azaborine | 8.26 | 8.32 | 8.43 | 8.40 | 8.30 | 8.37 | 6.64 | 8.51 | 8.46 | 8.6[11] |
| 1,3-azaborine | 7.79 | 7.90 | 8.00 | 7.98 | 8.00 | 8.10 | 6.32 | 8.30 | 8.24 | |
| 1,4-azaborine | 8.51 | 8.54 | 8.55 | 8.57 | 8.75 | 8.77 | 6.90 | 8.78 | 8.80 | |
| di-BN-doped benzene | 7.14 | 7.30 | 7.53 | 7.51 | 7.42 | 7.57 | 6.01 | 7.85 | 7.82 | |
| borazine | 9.94 | 9.94 | 9.88 | 9.92 | 9.95 | 9.95 | 8.19 | 9.89 | 9.92 | 9.88[12] |
| MSE(Exp.)† | −0.14 | −0.11 | −0.07 | −0.08 | −0.06 | −0.01 | −1.91 | −0.04 | −0.06 | |
| RMSE(Exp.)‡ | 0.21 | 0.18 | 0.10 | 0.12 | 0.18 | 0.16 | 1.92 | 0.06 | 0.10 | |
| MSE(CCSD(T))† | −0.07 | – | 0.08 | 0.07 | −0.07 | – | −1.81 | 0.07 | 0.05 | |
| RMSE(CCSD(T))‡ | 0.08 | – | 0.11 | 0.10 | 0.08 | – | 1.82 | 0.17 | 0.15 | |

† $\mathrm{MSE} = \frac{1}{N}\sum_i^N \left(E_i^{\mathrm{method}} - E_i^{\mathrm{ref}}\right)$, ‡ $\mathrm{RMSE} = \sqrt{\sum_i^N \frac{\left(E_i^{\mathrm{method}} - E_i^{\mathrm{ref}}\right)^2}{N}}$

Table S3: The ionization potentials (IPs) in eV for benzene and its mono-, di-, and tri-BN-doped derivatives are investigated using both conventional and pCCD-based methodologies with the cc-pVDZ basis set.

# S5 Calculated ionization potentials using cc-pVDZ and cc-pVTZ

| Molecule | **cc-pVDZ** IP-EOM- | | **cc-pVTZ** IP-EOM- | | Exp. |
|---|---|---|---|---|---|
| | fpCCD | fpCCSD | fpCCD | fpCCSD | |
| naphthalene | 8.18 | 8.07 | 8.39 | 8.26 | 8.14[13] |
| BN-1,2 naphthalene | 8.21 | 8.20 | 8.45 | 8.41 | 8.45[14] |
| BN-1,3-naphthalene | 7.77 | 7.69 | 8.01 | 7.91 | |
| BN-1,9 naphthalene | 7.76 | 7.70 | 8.01 | 7.93 | 7.78[14] |
| BN-2,7-naphthalene | 6.78 | 6.81 | 7.05 | 7.06 | |
| BN-9,1-naphthalene | 7.36 | 7.31 | 7.61 | 7.54 | 7.44[14] |
| BN-9,10-naphthalene | 8.26 | 8.16 | 8.50 | 8.37 | 8.42[14] |
| MSE(Exp.)† | −0.09 | −0.16 | 0.15 | 0.06 | |
| RMSE(Exp.)‡ | 0.14 | 0.18 | 0.17 | 0.10 | |

Table S4: Ionization potentials (IPs) in eV were calculated using partially relaxed geometries of mono-BN-doped naphthalene with cc-pVDZ and cc-pVTZ basis sets.

# S6 Naphthalene and its BN-doped isomers

| Molecule | Partially relaxed geometry | | | | | Exp. |
|---|---|---|---|---|---|---|
| | Standard Methods | | IP-EOM | | | |
| | CCSD | CCSD(T) | pCCD | fpCCD | fpCCSD | |
| naphthalene | 7.97 | 7.94 | 6.11 | 8.18 | 8.07 | 8.14 [13] |
| BN-1,2-naphthalene | 8.19 | 8.27 | 6.07 | 8.21 | 8.20 | 8.45 [14] |
| BN-1,3-naphthalene | 7.48 | 7.55 | 5.69 | 7.77 | 7.69 | – |
| BN-1,4-naphthalene | 7.98 | 7.93 | 6.01 | 8.06 | 8.01 | – |
| BN-1,5-naphthalene | 6.70 | 6.79 | 4.76 | 6.85 | 6.84 | – |
| BN-1,6-naphthalene | 6.82 | 6.95 | 4.72 | 6.82 | 6.88 | – |
| BN-1,7-naphthalene | 7.14 | 7.23 | 5.22 | 7.29 | 7.27 | – |
| BN-1,8-naphthalene | 6.69 | 6.80 | 4.73 | 6.72 | 6.77 | – |
| BN-1,9-naphthalene | 7.49 | 7.55 | 5.68 | 7.76 | 7.70 | 7.78 [14] |
| BN-1,10-naphthalene | 7.49 | 7.56 | 5.46 | 7.64 | 7.62 | – |
| BN-2,1-naphthalene | 7.77 | 7.77 | 5.92 | 7.96 | 7.88 | – |
| BN-2,3-naphthalene | 7.24 | 7.31 | 5.30 | 7.40 | 7.36 | – |
| BN-2,4-naphthalene | 7.21 | 7.30 | 5.39 | 7.50 | 7.43 | – |
| BN-2,5-naphthalene | 6.57 | 6.66 | 4.79 | 6.86 | 6.80 | – |
| BN-2,6-naphthalene | 7.10 | 7.18 | 5.26 | 7.32 | 7.28 | – |
| BN-2,7-naphthalene | 6.73 | 6.86 | 4.68 | 6.78 | 6.81 | – |
| BN-2,8-naphthalene | 6.67 | 6.75 | 4.80 | 6.90 | 6.85 | – |
| BN-2,9-naphthalene | 7.66 | 7.66 | 5.62 | 7.80 | 7.76 | – |
| BN-3,9-naphthalene | 7.42 | 7.46 | 5.70 | 7.73 | 7.65 | – |
| BN-9,1-naphthalene | 7.18 | 7.25 | 5.29 | 7.36 | 7.31 | 7.44 [14] |
| BN-9,2-naphthalene | 7.33 | 7.44 | 5.33 | 7.41 | 7.43 | – |
| BN-9,3-naphthalene | 7.51 | 7.56 | 5.61 | 7.67 | 7.65 | – |
| BN-10,1-naphthalene | 7.10 | 7.18 | 5.31 | 7.36 | 7.30 | – |
| BN-9,10-naphthalene | 8.02 | 7.97 | 6.29 | 8.26 | 8.16 | 8.42 [14] |
| MSE(Exp.)$^{\dagger}$ | −0.28 | −0.25 | −2.16 | −0.09 | −0.16 | |
| RMSE(Exp.)$^{\ddagger}$ | 0.29 | 0.27 | 2.16 | 0.14 | 0.18 | |
| MSE(CCSD(T))$^{\dagger}$ | −0.06 | – | −1.97 | 0.11 | 0.08 | |
| RMSE(CCSD(T))$^{\ddagger}$ | 0.08 | – | 1.97 | 0.16 | 0.11 | |

Table S5: Ionization potentials (IPs) in eV of naphthalene and 23 mono-BN-doped naphthalene isomers using partially relaxed geometry with the cc-pVDZ basis set. Mean signed error (MSE) and root mean square error (RMSE) with respect to the experiment and CCSD(T) results.

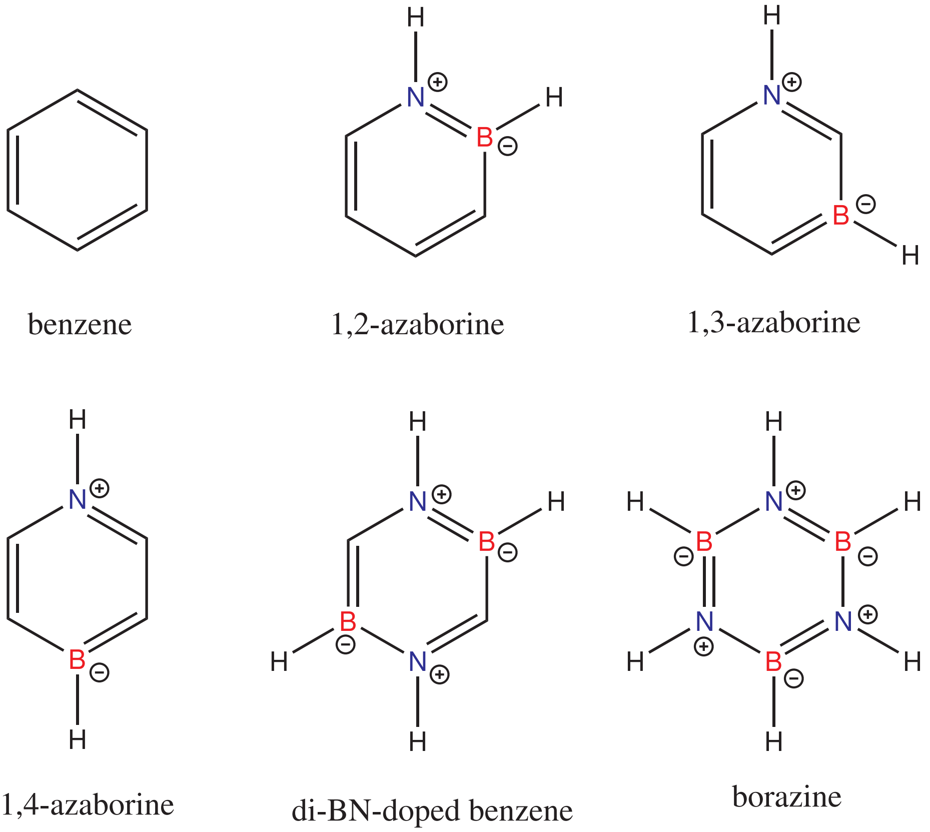


Figure S1: The structures of benzene and its mono-, di-, and tri-BN-doped derivatives in distinct confugurations.

## S7 Relative energy comparison: BN-naphthalene vs BN-benzene

**Neutral**

| BN-naphthalene Position | BN-naphthalene Relative Energy | Average | BN-benzene Relative Energy | BN-benzene Position |
|---|---|---|---|---|
| (2,1) | -8.7 | 0.0 | 0.0 | (1,2) |
| (1,2) | -8.6 | | | |
| (9,10) | -0.1 | | | |
| (1,9) | 0.0 | | | |
| (2,3) | 6.0 | | | |
| (9,1) | 11.4 | | | |
| (1,4) | 27.9 | 33.4 | 33.2 | (1,4) |
| (3,9) | 30.9 | | | |
| (9,3) | 41.5 | | | |
| (2,9) | 33.9 | 38.5 | 39.6 | (1,3) |
| (1,3) | 36.8 | | | |
| (2,4) | 37.6 | | | |
| (1,10) | 33.8 | | | |
| (10,1) | 43.4 | | | |
| (9,2) | 45.3 | | | |

**Cation**

| BN-naphthalene Position | BN-naphthalene Relative Energy | Average | BN-benzene Relative Energy | BN-benzene Position |
|---|---|---|---|---|
| (2,1) | -6.2 | 0.0 | 0.0 | (1,2) |
| (2,3) | -3.3 | | | |
| (1,9) | -1.6 | | | |
| (9,1) | 0.9 | | | |
| (1,2) | 1.3 | | | |
| (9,10) | 8.9 | | | |
| (3,9) | 28.2 | 33.5 | 40.8 | (1,4) |
| (1,4) | 33.6 | | | |
| (9,3) | 38.8 | | | |
| (2,4) | 29.8 | 33.2 | 34.4 | (1,3) |
| (1,10) | 30.4 | | | |
| (10,1) | 32.6 | | | |
| (2,9) | 33.7 | | | |
| (1,3) | 35.1 | | | |
| (9,2) | 37.6 | | | |

Table S6: Two tables (the left for neutral and the right for cation) provide a comparative analysis of average relative energies (in kcal/mol) of three benzene-based isomers: ortho (BN-1,2 benzene), meta (BN-1,3 benzene), and para (BN-1,4 benzene), with their corresponding naphthalene-based isomers: ortho-like (BN-1,2 naph), meta-like (BN-1,3 naph), and para-like (BN-1,4 naph).

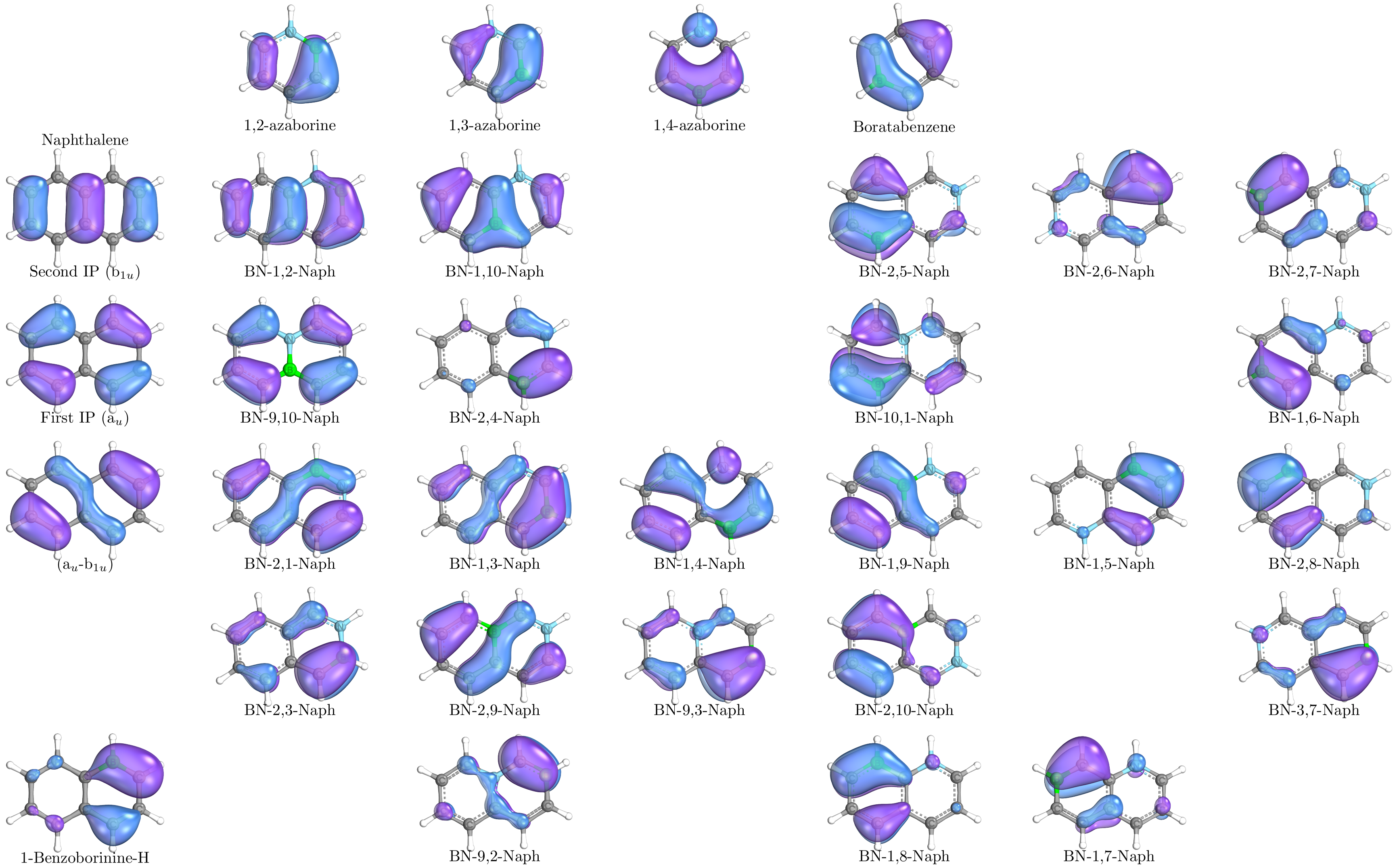


Figure S2: Singly occupied molecular orbitals (SOMOs) for the cation ground states of all mono-BN-doped benzene and naphthalene isomers, and naphthalene. SOMOs for neutral Boratebenzene and protonated 1-Benzoborinine were also included for comparison. All orbitals are obtained via the canonical RHF ones, using the cc-pVDZ basis set.

(a) BN-1,6-Naphtalene
Mono-anion SOMO

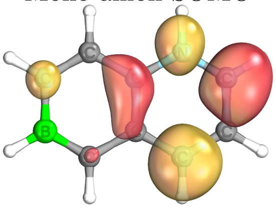

(b) Neutral HOMO and LUMO

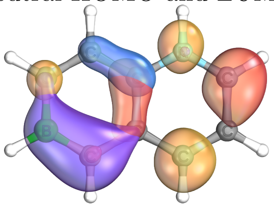

(c) Mono-cation SOMO

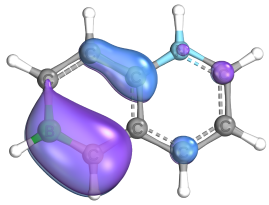

Figure S3: Singly occupied molecular orbital (SOMO) of the BN-1,6 naphthalene isomer, categorized into three parts: (a) the top part denotes the mono-anion; (b) the middle part represents the neutral first excited singlet state; and (c) the bottom part corresponds to the mono-cation.

# S8 Fully relaxed geometries: benzene, naphthalene and its BN-doped derivatives

All structures were optimized using density functional theory (DFT) with the exchange-correlational functional BP86 and the def2-TZVP basis set in the Turbomole V7.3 software package, where atomic coordinates are expressed in angstroms (Å).

benzene

| | | | |
|---|---|---|---|
| C | 0.6856851 | −0.0928977 | 1.2145255 |
| C | −0.7109053 | −0.0354118 | 1.2030161 |
| C | −1.3966066 | 0.0574633 | −0.0115132 |
| C | −0.6857037 | 0.0928625 | −1.2145391 |
| C | 0.7108924 | 0.0353794 | −1.2030296 |
| C | 1.3965921 | −0.0574958 | 0.0115045 |
| H | 1.2210049 | −0.1653856 | 2.1627202 |
| H | −1.2658986 | −0.0630379 | 2.1422241 |
| H | −2.4869279 | 0.1023295 | −0.0205034 |
| H | −1.2210184 | 0.1653456 | −2.1627412 |
| H | 1.2658827 | 0.0630080 | −2.1422449 |
| H | 2.4869132 | −0.1023595 | 0.0204911 |

1,2 azaborine

| | | | |
|---|---|---|---|
| N | 0.1894482 | −1.3670459 | 0.0009848 |
| B | −1.1742457 | −0.8934590 | 0.0005737 |
| C | −1.3172455 | 0.6128926 | −0.0001999 |
| C | −0.1779130 | 1.3952711 | −0.0005083 |
| C | 1.1232041 | 0.8226049 | −0.0000695 |
| C | 1.2793182 | −0.5416733 | 0.0006675 |
| H | 0.3924591 | −2.3637464 | 0.0015248 |
| H | −2.0510118 | −1.7137300 | 0.0008820 |
| H | −2.2860488 | 1.1183270 | −0.0005327 |
| H | −0.2505048 | 2.4876789 | −0.0010616 |
| H | 2.0095929 | 1.4558628 | −0.0002949 |
| H | 2.2629471 | −1.0129828 | 0.0010340 |

1,3 azaborine

| | | | |
|---|---|---|---|
| N | 0.1636102 | −1.3376339 | 0.0009586 |
| C | −1.1193926 | −0.8860868 | 0.0005614 |
| B | −1.3894137 | 0.5959655 | −0.0002070 |

| | | | |
|---|---|---|---|
| C | −0.1346221 | 1.4407891 | −0.0005409 |
| C | 1.1258233 | 0.8381503 | −0.0000796 |
| C | 1.2661800 | −0.5469051 | 0.0006618 |
| H | 0.3265393 | −2.3438081 | 0.0015285 |
| H | −1.8524337 | −1.6960282 | 0.0008965 |
| H | −2.5102362 | 1.0309211 | −0.0004666 |
| H | −0.1490159 | 2.5357806 | −0.0010956 |
| H | 2.0467997 | 1.4272294 | −0.0002609 |
| H | 2.2261617 | −1.0583740 | 0.0010437 |

1,4 azaborine

| | | | |
|---|---|---|---|
| N | 0.1587850 | −1.3283709 | 0.0009530 |
| C | −1.1174548 | −0.8264541 | 0.0005528 |
| C | −1.3544528 | 0.5232374 | −0.0001714 |
| B | −0.1785231 | 1.4932750 | −0.0005747 |
| C | 1.1929068 | 0.8278011 | −0.0000596 |
| C | 1.2808365 | −0.5397420 | 0.0006615 |
| H | 0.2792433 | −2.3355976 | 0.0015260 |
| H | −1.8964720 | −1.5923900 | 0.0008650 |
| H | −2.4053926 | 0.8230749 | −0.0003890 |
| H | −0.3214432 | 2.6886661 | −0.0012221 |
| H | 2.1435640 | 1.3669244 | −0.0001956 |
| H | 2.2184030 | −1.1004242 | 0.0010539 |

di-BN-doped benzene

| | | | |
|---|---|---|---|
| N | 0.1869879 | −1.3740388 | 0.0009770 |
| B | −1.1766783 | −0.8931830 | 0.0005694 |
| C | −1.3059189 | 0.5995661 | −0.0001991 |
| N | −0.1871774 | 1.3740511 | −0.0004877 |
| B | 1.1765709 | 0.8932386 | −0.0000873 |
| C | 1.3059069 | −0.5995543 | 0.0006841 |
| H | 0.3443910 | −2.3827157 | 0.0015275 |
| H | −2.0755167 | −1.6849869 | 0.0008694 |
| H | −2.2522389 | 1.1448072 | −0.0005564 |
| H | −0.3443421 | 2.3826280 | −0.0009935 |
| H | 2.0755814 | 1.6850814 | −0.0003837 |
| H | 2.2524341 | −1.1448938 | 0.0010804 |

borazine

| | | | |
|---|---|---|---|
| N | 0.1675586 | −1.4015476 | 0.0008222 |
| B | −1.1664475 | −0.8731781 | 0.0005093 |
| N | −1.2975446 | 0.5556557 | −0.0001741 |

| | | | |
|---|---|---|---|
| B | −0.1729661 | 1.4467640 | −0.0005519 |
| N | 1.1299946 | 0.8458924 | −0.0000718 |
| B | 1.3394101 | −0.5735817 | 0.0006298 |
| H | 0.2880738 | −2.4095137 | 0.0016638 |
| H | −2.1286259 | −1.5934348 | 0.0008658 |
| H | −2.2307191 | 0.9552705 | −0.0003575 |
| H | −0.3156523 | 2.6401615 | −0.0012341 |
| H | 1.9426626 | 1.4542320 | −0.0001870 |
| H | 2.4442557 | −1.0467203 | 0.0010854 |

naphthalene

| | | | |
|---|---|---|---|
| C | −1.2447920 | −1.4038958 | 0.0010519 |
| C | −2.4360661 | −0.7081483 | 0.0002546 |
| C | −2.4360428 | 0.7081643 | −0.0004050 |
| C | −1.2448037 | 1.4038952 | −0.0001649 |
| C | 0.0000067 | 0.7189067 | 0.0001280 |
| C | 1.2448253 | 1.4038898 | −0.0000388 |
| C | 2.4360546 | 0.7081538 | −0.0001991 |
| C | 2.4360771 | −0.7081570 | −0.0002948 |
| C | 1.2447941 | −1.4039018 | 0.0001276 |
| C | 0.0000050 | −0.7188880 | 0.0005166 |
| H | −1.2427061 | −2.4962119 | 0.0020359 |
| H | −3.3844484 | −1.2476265 | 0.0002216 |
| H | −3.3844539 | 1.2476326 | −0.0009898 |
| H | −1.2427043 | 2.4962144 | −0.0002964 |
| H | 1.2427229 | 2.4962093 | −0.0001908 |
| H | 3.3844711 | 1.2476186 | −0.0002617 |
| H | 3.3844531 | −1.2476401 | −0.0007464 |
| H | 1.2427072 | −2.4962154 | −0.0001483 |

BN-1,2 naphthalene

| | | | |
|---|---|---|---|
| N | −1.2034592 | −1.3851988 | 0.0008305 |
| B | −2.4867970 | −0.7542254 | 0.0002847 |
| C | −2.4640232 | 0.7715226 | −0.0003652 |
| C | −1.2564300 | 1.4167052 | −0.0001353 |
| C | −0.0028130 | 0.7118884 | 0.0002272 |
| C | 1.2387315 | 1.3883063 | −0.0000038 |
| C | 2.4391319 | 0.6963277 | −0.0002102 |
| C | 2.4317673 | −0.7112367 | −0.0002135 |
| C | 1.2321052 | −1.4072669 | 0.0002090 |
| C | 0.0058200 | −0.7134151 | 0.0005029 |
| H | −1.1239895 | −2.4003194 | 0.0013315 |

| | | | |
|---|---|---|---|
| H | −3.4592287 | −1.4580497 | 0.0004199 |
| H | −3.3733181 | 1.3773675 | −0.0008922 |
| H | −1.1965612 | 2.5109592 | −0.0003698 |
| H | 1.2323650 | 2.4805862 | −0.0001382 |
| H | 3.3860286 | 1.2370512 | −0.0004476 |
| H | 3.3741896 | −1.2608457 | −0.0005833 |
| H | 1.2265807 | −2.5001567 | 0.0001533 |

BN-1,3 naphthalene

| | | | |
|---|---|---|---|
| N | −1.2005483 | −1.3428242 | 0.0007474 |
| C | −2.4190229 | −0.7569942 | 0.0003276 |
| B | −2.5527135 | 0.7500890 | −0.0003833 |
| C | −1.2354614 | 1.4632224 | −0.0001387 |
| C | −0.0087265 | 0.7595669 | 0.0001881 |
| C | 1.2703974 | 1.3982811 | 0.0000186 |
| C | 2.4438591 | 0.6775456 | −0.0002091 |
| C | 2.4171498 | −0.7397644 | −0.0002226 |
| C | 1.2091066 | −1.4063305 | 0.0001916 |
| C | 0.0006795 | −0.6751690 | 0.0004510 |
| H | −1.1376728 | −2.3613243 | 0.0012032 |
| H | −3.2346703 | −1.4850202 | 0.0006427 |
| H | −3.6316386 | 1.2795720 | −0.0010256 |
| H | −1.1422227 | 2.5553803 | −0.0002781 |
| H | 1.2920319 | 2.4899296 | −0.0000728 |
| H | 3.4036416 | 1.1963345 | −0.0004287 |
| H | 3.3505273 | −1.3033665 | −0.0005741 |
| H | 1.1753837 | −2.4991280 | 0.0001630 |

BN-1,4 naphthalene

| | | | |
|---|---|---|---|
| N | −1.1974829 | −1.3209271 | 0.0008173 |
| C | −2.4147682 | −0.7013298 | 0.0003066 |
| C | −2.5513507 | 0.6647501 | −0.0003630 |
| B | −1.3052309 | 1.5272653 | −0.0001375 |
| C | 0.0312719 | 0.7642716 | 0.0002457 |
| C | 1.3051003 | 1.3857824 | 0.0000065 |
| C | 2.4833546 | 0.6554044 | −0.0002170 |
| C | 2.4320264 | −0.7528532 | −0.0002298 |
| C | 1.2133529 | −1.4107794 | 0.0001723 |
| C | 0.0194902 | −0.6585894 | 0.0004699 |
| H | −1.1738670 | −2.3361447 | 0.0012393 |
| H | −3.2583232 | −1.3963521 | 0.0005029 |
| H | −3.5753744 | 1.0438749 | −0.0008966 |

| | | | |
|---|---|---|---|
| H | −1.3269716 | 2.7317204 | −0.0003077 |
| H | 1.3400063 | 2.4774109 | −0.0000923 |
| H | 3.4493440 | 1.1622379 | −0.0004420 |
| H | 3.3567793 | −1.3322826 | −0.0005813 |
| H | 1.1727430 | −2.5034596 | 0.0001067 |

BN-1,5 naphthalene

| | | | |
|---|---|---|---|
| N | −1.2287883 | −1.3472719 | 0.0008370 |
| C | −2.4263707 | −0.7273722 | 0.0003524 |
| C | −2.4953514 | 0.6532445 | −0.0003569 |
| C | −1.2896146 | 1.3809033 | −0.0002147 |
| C | −0.0366704 | 0.7679707 | 0.0002191 |
| B | 1.3043384 | 1.5349454 | 0.0000726 |
| C | 2.5391628 | 0.6747868 | −0.0001551 |
| C | 2.4198914 | −0.7155466 | −0.0002468 |
| C | 1.1848305 | −1.4004032 | 0.0001370 |
| C | −0.0052023 | −0.6844026 | 0.0004313 |
| H | −1.2058198 | −2.3654354 | 0.0013131 |
| H | −3.3031506 | −1.3733803 | 0.0006103 |
| H | −3.4670166 | 1.1431323 | −0.0008991 |
| H | −1.3269245 | 2.4732302 | −0.0004796 |
| H | 1.2965005 | 2.7420632 | −0.0000521 |
| H | 3.5567837 | 1.0768383 | −0.0004081 |
| H | 3.3202871 | −1.3401190 | −0.0006354 |
| H | 1.1632149 | −2.4931835 | 0.0000751 |

BN-1,6 naphthalene

| | | | |
|---|---|---|---|
| N | −1.2025219 | −1.3188569 | 0.0007384 |
| C | −2.4085837 | −0.7176722 | 0.0003639 |
| C | −2.4598813 | 0.6773537 | −0.0002472 |
| C | −1.2771429 | 1.4015625 | −0.0002156 |
| C | 0.0046461 | 0.7826023 | 0.0001571 |
| C | 1.2228665 | 1.4784166 | 0.0000384 |
| B | 2.5311116 | 0.7403142 | −0.0001137 |
| C | 2.4252413 | −0.7956522 | −0.0002321 |
| C | 1.1980546 | −1.4301713 | 0.0001726 |
| C | 0.0070640 | −0.6744757 | 0.0004264 |
| H | −1.1683488 | −2.3381919 | 0.0011202 |
| H | −3.2818861 | −1.3650015 | 0.0005831 |
| H | −3.4294131 | 1.1730141 | −0.0007315 |
| H | −1.3136358 | 2.4931339 | −0.0005594 |
| H | 1.1376820 | 2.5708392 | −0.0001333 |

| | | | |
|---|---|---|---|
| H | 3.6022121 | 1.2950037 | −0.0002502 |
| H | 3.3054134 | −1.4478620 | −0.0006603 |
| H | 1.1072219 | −2.5243564 | 0.0001435 |

BN-1,7 naphthalene

| | | | |
|---|---|---|---|
| N | −1.2436530 | −1.3392062 | 0.0009163 |
| C | −2.4264104 | −0.6851709 | 0.0003347 |
| C | −2.4526197 | 0.6944842 | −0.0003569 |
| C | −1.2271075 | 1.3854221 | −0.0001860 |
| C | −0.0021716 | 0.7237045 | 0.0001892 |
| C | 1.2451792 | 1.4285613 | 0.0000440 |
| C | 2.4483358 | 0.7709462 | −0.0001302 |
| B | 2.5083145 | −0.7707244 | −0.0003176 |
| C | 1.1779737 | −1.4832114 | 0.0001159 |
| C | 0.0137656 | −0.7351491 | 0.0004367 |
| H | −1.2456234 | −2.3572904 | 0.0015470 |
| H | −3.3217786 | −1.3043856 | 0.0004668 |
| H | −3.4056563 | 1.2183257 | −0.0009413 |
| H | −1.2252352 | 2.4778551 | −0.0004267 |
| H | 1.1820138 | 2.5226057 | −0.0000612 |
| H | 3.3490231 | 1.3942736 | −0.0002489 |
| H | 3.5568862 | −1.3658351 | −0.0008411 |
| H | 1.0688637 | −2.5752052 | 0.0000594 |

BN-1,8 naphthalene

| | | | |
|---|---|---|---|
| N | −1.2600920 | −1.3114963 | 0.0008750 |
| C | −2.4422177 | −0.6783041 | 0.0003155 |
| C | −2.4608332 | 0.7200453 | −0.0003283 |
| C | −1.2560229 | 1.4096454 | −0.0001353 |
| C | −0.0119813 | 0.7358727 | 0.0002354 |
| C | 1.2289698 | 1.4064483 | 0.0000337 |
| C | 2.4294082 | 0.6944976 | −0.0001709 |
| C | 2.5029057 | −0.7138011 | −0.0002886 |
| B | 1.2589943 | −1.5432147 | 0.0001697 |
| C | −0.0201347 | −0.7169044 | 0.0005559 |
| H | −1.2476869 | −2.3326503 | 0.0015070 |
| H | −3.3378027 | −1.2962809 | 0.0003622 |
| H | −3.4164008 | 1.2411375 | −0.0009033 |
| H | −1.2613465 | 2.5025651 | −0.0003713 |
| H | 1.2336739 | 2.4980737 | −0.0001588 |
| H | 3.3551412 | 1.2804027 | −0.0003469 |
| H | 3.5096253 | −1.1433965 | −0.0007363 |

| | | | |
|---|---|---|---|
| H | 1.1959002 | −2.7526398 | −0.0000145 |

BN-1,9 naphthalene

| | | | |
|---|---|---|---|
| N | −1.3206558 | −1.3989441 | 0.0008343 |
| C | −2.4708732 | −0.6827215 | 0.0003961 |
| C | −2.4551683 | 0.7051600 | −0.0002234 |
| C | −1.2433533 | 1.4184774 | −0.0002164 |
| C | −0.0107520 | 0.7555801 | 0.0001683 |
| C | 1.2490033 | 1.4277869 | −0.0000146 |
| C | 2.4244007 | 0.7100692 | −0.0002152 |
| C | 2.4621242 | −0.7250046 | −0.0002520 |
| C | 1.3283197 | −1.5079521 | 0.0002186 |
| B | −0.0052019 | −0.7797391 | 0.0005145 |
| H | −1.4251878 | −2.4119633 | 0.0012550 |
| H | −3.4091722 | −1.2391868 | 0.0005534 |
| H | −3.4088629 | 1.2318596 | −0.0006949 |
| H | −1.2835947 | 2.5140865 | −0.0005766 |
| H | 1.2834854 | 2.5225332 | −0.0001139 |
| H | 3.3759329 | 1.2463988 | −0.0004305 |
| H | 3.4530367 | −1.1908526 | −0.0007001 |
| H | 1.4566192 | −2.5955875 | 0.0000973 |

BN-1,10 naphthalene

| | | | |
|---|---|---|---|
| N | −1.2085912 | −1.3425422 | 0.0007137 |
| C | −2.4103014 | −0.7245260 | 0.0003513 |
| C | −2.5121083 | 0.6692142 | −0.0002176 |
| C | −1.3791997 | 1.4837372 | −0.0001652 |
| B | −0.0020623 | 0.8517777 | 0.0002087 |
| C | 1.3841977 | 1.4926970 | 0.0000120 |
| C | 2.4989279 | 0.6792774 | −0.0002358 |
| C | 2.4088025 | −0.7468455 | −0.0002001 |
| C | 1.2027232 | −1.4240079 | 0.0002157 |
| C | −0.0021739 | −0.6859866 | 0.0004551 |
| H | −1.2054511 | −2.3643168 | 0.0010345 |
| H | −3.2813773 | −1.3776370 | 0.0005508 |
| H | −3.5229167 | 1.0841582 | −0.0006776 |
| H | −1.5521516 | 2.5654126 | −0.0005333 |
| H | 1.5485814 | 2.5754589 | −0.0000790 |
| H | 3.5053777 | 1.1100625 | −0.0004919 |
| H | 3.3350509 | −1.3254510 | −0.0005468 |
| H | 1.1927723 | −2.5204827 | 0.000205 |

BN-2,1 naphthalene

| | | | |
|---|---|---|---|
| B | −1.2961047 | −1.4874370 | 0.0009445 |
| N | −2.4679419 | −0.6688837 | 0.0004070 |
| C | −2.4264582 | 0.7124602 | −0.0002639 |
| C | −1.2418922 | 1.3848772 | −0.0002449 |
| C | 0.0199744 | 0.6944527 | 0.0001534 |
| C | 1.2441731 | 1.4056631 | −0.0000517 |
| C | 2.4527791 | 0.7278427 | −0.0002746 |
| C | 2.4820092 | −0.6805812 | −0.0002993 |
| C | 1.2913443 | −1.3932069 | 0.0001980 |
| C | 0.0383801 | −0.7382365 | 0.0005005 |
| H | −1.4272740 | −2.6814117 | 0.0016482 |
| H | −3.3967033 | −1.0812178 | 0.0004606 |
| H | −3.3843345 | 1.2333864 | −0.0008161 |
| H | −1.2550826 | 2.4752061 | −0.0006907 |
| H | 1.2270122 | 2.4981236 | −0.0001464 |
| H | 3.3893801 | 1.2887719 | −0.0004787 |
| H | 3.4394056 | −1.2041491 | −0.0006842 |
| H | 1.3114333 | −2.4856600 | 0.0002383 |

BN-2,3 naphthalene

| | | | |
|---|---|---|---|
| C | −1.2197888 | −1.3817956 | 0.0007725 |
| N | −2.3976248 | −0.7228054 | 0.0003866 |
| B | −2.5253353 | 0.7294171 | −0.0002875 |
| C | −1.2230697 | 1.4630173 | −0.0002046 |
| C | −0.0084912 | 0.7594111 | 0.0001269 |
| C | 1.2711322 | 1.4087459 | −0.0000264 |
| C | 2.4413702 | 0.6925595 | −0.0002317 |
| C | 2.4286744 | −0.7342852 | −0.0002240 |
| C | 1.2340377 | −1.4070695 | 0.0002213 |
| C | −0.0060006 | −0.6953343 | 0.0004554 |
| H | −1.2447729 | −2.4732681 | 0.0012280 |
| H | −3.2289747 | −1.3102812 | 0.0005875 |
| H | −3.6364763 | 1.1825653 | −0.0008637 |
| H | −1.1569723 | 2.5548341 | −0.0004591 |
| H | 1.2907584 | 2.5005915 | −0.0001053 |
| H | 3.3991303 | 1.2163832 | −0.0004321 |
| H | 3.3710285 | −1.2832254 | −0.0005807 |
| H | 1.2114747 | −2.4994602 | 0.0002369 |

BN-2,4 naphthalene

| | | | |
|---|---|---|---|
| C | −1.2187702 | −1.3626379 | 0.0007809 |
| N | −2.3829502 | −0.6895606 | 0.0003687 |
| C | −2.5247526 | 0.6728636 | −0.0002712 |
| B | −1.2980857 | 1.5302272 | −0.0001889 |
| C | 0.0281645 | 0.7632270 | 0.0002100 |
| C | 1.3011076 | 1.3933577 | −0.0000006 |
| C | 2.4784089 | 0.6677722 | −0.0002236 |
| C | 2.4431231 | −0.7479169 | −0.0002388 |
| C | 1.2355643 | −1.4115425 | 0.0002000 |
| C | 0.0140374 | −0.6776949 | 0.0004658 |
| H | −1.2840646 | −2.4494085 | 0.0012224 |
| H | −3.2280055 | −1.2599030 | 0.0005758 |
| H | −3.5685087 | 0.9901208 | −0.0008021 |
| H | −1.3536164 | 2.7320214 | −0.0005179 |
| H | 1.3353567 | 2.4851064 | −0.0001007 |
| H | 3.4418026 | 1.1811832 | −0.0004450 |
| H | 3.3766189 | −1.3133362 | −0.0006163 |
| H | 1.2046699 | −2.5038788 | 0.0001814 |

BN-2,5 naphthalene

| | | | |
|---|---|---|---|
| C | −1.2285414 | −1.3751514 | 0.0007645 |
| N | −2.3962616 | −0.7088788 | 0.0004196 |
| C | −2.4768535 | 0.6675299 | −0.0002512 |
| C | −1.3030632 | 1.3865893 | −0.0002769 |
| C | −0.0301791 | 0.7716174 | 0.0001308 |
| B | 1.2930003 | 1.5394858 | 0.0000302 |
| C | 2.5262074 | 0.6787671 | −0.0001723 |
| C | 2.4302981 | −0.7256150 | −0.0002427 |
| C | 1.2136729 | −1.4068745 | 0.0001708 |
| C | −0.0071200 | −0.6927511 | 0.0003864 |
| H | −1.2950249 | −2.4638144 | 0.0012427 |
| H | −3.2597019 | −1.2451111 | 0.0006946 |
| H | −3.4770738 | 1.0921282 | −0.0007399 |
| H | −1.3625497 | 2.4763587 | −0.0006660 |
| H | 1.2991321 | 2.7476930 | −0.0000665 |
| H | 3.5414996 | 1.0894680 | −0.0004058 |
| H | 3.3423826 | −1.3325609 | −0.0006143 |
| H | 1.1902760 | −2.4988802 | 0.0001958 |

BN-2,6 naphthalene

| | | | |
|---|---|---|---|
| C | −1.2077761 | −1.3579009 | 0.0007566 |
| N | −2.3916978 | −0.6917825 | 0.0004476 |

| | | | |
|---|---|---|---|
| C | −2.4434812 | 0.6886452 | −0.0001748 |
| C | −1.2855880 | 1.4052887 | −0.0002511 |
| C | 0.0080165 | 0.7759382 | 0.0001064 |
| C | 1.2075735 | 1.4772602 | −0.0000229 |
| B | 2.5305636 | 0.7412642 | −0.0001493 |
| C | 2.4480408 | −0.7978445 | −0.0002707 |
| C | 1.2326734 | −1.4308738 | 0.0001887 |
| C | 0.0027143 | −0.6919577 | 0.0004246 |
| H | −1.2685883 | −2.4464375 | 0.0011604 |
| H | −3.2573385 | −1.2200898 | 0.0005601 |
| H | −3.4373857 | 1.1304613 | −0.0006322 |
| H | −1.3416162 | 2.4943955 | −0.0006939 |
| H | 1.1253540 | 2.5702637 | −0.0001848 |
| H | 3.5913124 | 1.3159135 | −0.0002166 |
| H | 3.3368864 | −1.4379899 | −0.0006769 |
| H | 1.1504367 | −2.5245539 | 0.0002288 |

BN-2,7 naphthalene

| | | | |
|---|---|---|---|
| C | −1.2499832 | −1.3696999 | 0.0008311 |
| N | −2.3962289 | −0.6653614 | 0.0004080 |
| C | −2.4362183 | 0.7082745 | −0.0002633 |
| C | −1.2413039 | 1.3919736 | −0.0002615 |
| C | 0.0011802 | 0.7240393 | 0.0001255 |
| C | 1.2315030 | 1.4259300 | 0.0000150 |
| C | 2.4460428 | 0.7635937 | −0.0001678 |
| B | 2.5305591 | −0.7701592 | −0.0003410 |
| C | 1.2082333 | −1.4824152 | 0.0001552 |
| C | 0.0104687 | −0.7444472 | 0.0004027 |
| H | −1.3503434 | −2.4549634 | 0.0013831 |
| H | −3.2760850 | −1.1748715 | 0.0006070 |
| H | −3.4198360 | 1.1684383 | −0.0007757 |
| H | −1.2621005 | 2.4827654 | −0.0006505 |
| H | 1.1774068 | 2.5207421 | −0.0000343 |
| H | 3.3400522 | 1.3981072 | −0.0002510 |
| H | 3.5894086 | −1.3485403 | −0.0008282 |
| H | 1.0973445 | −2.5734060 | 0.0002457 |

BN-2,8 naphthalene

| | | | |
|---|---|---|---|
| C | −1.2665971 | −1.3526243 | 0.0007533 |
| N | −2.4302234 | −0.6534230 | 0.0004240 |
| C | −2.4454311 | 0.7304226 | −0.0001605 |
| C | −1.2668347 | 1.4149931 | −0.0001887 |

| | | | |
|---|---|---|---|
| C | −0.0089930 | 0.7297473 | 0.0001838 |
| C | 1.2147080 | 1.3954233 | −0.0000096 |
| C | 2.4388468 | 0.6888843 | −0.0002243 |
| C | 2.5361970 | −0.7023993 | −0.0002791 |
| B | 1.2831055 | −1.5399588 | 0.0002353 |
| C | −0.0310288 | −0.7343513 | 0.0005023 |
| H | −1.3661505 | −2.4392141 | 0.0010981 |
| H | −3.3129003 | −1.1547364 | 0.0005033 |
| H | −3.4263852 | 1.1996865 | −0.0006405 |
| H | −1.2936364 | 2.5057554 | −0.0006396 |
| H | 1.2244050 | 2.4877488 | −0.0001652 |
| H | 3.3520544 | 1.2952634 | −0.0004457 |
| H | 3.5462993 | −1.1235455 | −0.0006504 |
| H | 1.2526645 | −2.7476719 | 0.0003035 |

BN-2,9 naphthalene

| | | | |
|---|---|---|---|
| C | −1.3565293 | −1.4492865 | 0.0008623 |
| N | −2.4561219 | −0.6579890 | 0.0004597 |
| C | −2.4375968 | 0.7142845 | −0.0001844 |
| C | −1.2458933 | 1.4074072 | −0.0002611 |
| C | −0.0029091 | 0.7316056 | 0.0001333 |
| C | 1.2393389 | 1.4218660 | −0.0000401 |
| C | 2.4301257 | 0.7194128 | −0.0002365 |
| C | 2.4967556 | −0.7090566 | −0.0002881 |
| C | 1.3676134 | −1.5018920 | 0.0001898 |
| B | 0.0093883 | −0.8088342 | 0.0005008 |
| H | −1.5938074 | −2.5166122 | 0.0013725 |
| H | −3.3761403 | −1.0949516 | 0.0006402 |
| H | −3.4129647 | 1.1960574 | −0.0006440 |
| H | −1.2984761 | 2.5008494 | −0.0007181 |
| H | 1.2626905 | 2.5174180 | −0.0001290 |
| H | 3.3705009 | 1.2763900 | −0.0004325 |
| H | 3.4952029 | −1.1586164 | −0.0007242 |
| H | 1.5089226 | −2.5880524 | 0.0000995 |

BN-3,9 naphthalene

| | | | |
|---|---|---|---|
| C | −1.3846544 | −1.4795975 | 0.0009718 |
| C | −2.4963931 | −0.6829155 | 0.0004864 |
| N | −2.3930617 | 0.6948478 | −0.0002060 |
| C | −1.2193312 | 1.3806946 | −0.0002611 |
| C | −0.0027684 | 0.7184052 | 0.0001779 |
| C | 1.2386999 | 1.4322652 | −0.0000249 |

| | | | |
|---|---|---|---|
| C | 2.4268102 | 0.7436004 | −0.0002400 |
| C | 2.4928454 | −0.6925806 | −0.0003085 |
| C | 1.3753452 | −1.4952910 | 0.0001718 |
| B | −0.0018828 | −0.8302295 | 0.0005541 |
| H | −1.5676399 | −2.5577678 | 0.0014891 |
| H | −3.5257983 | −1.0459988 | 0.0005398 |
| H | −3.2551679 | 1.2306807 | −0.0007564 |
| H | −1.3245367 | 2.4696891 | −0.0007429 |
| H | 1.2466425 | 2.5278341 | −0.0001084 |
| H | 3.3656041 | 1.3017971 | −0.0004393 |
| H | 3.4946629 | −1.1359452 | −0.0007641 |
| H | 1.5307240 | −2.5794884 | 0.0000608 |

BN-9,1 naphthalene

| | | | |
|---|---|---|---|
| B | −1.2313286 | −1.4748848 | 0.0011195 |
| C | −2.5030482 | −0.6952303 | 0.0001912 |
| C | −2.4432807 | 0.6975405 | −0.0004162 |
| C | −1.2220015 | 1.3818680 | −0.0002055 |
| C | 0.0051792 | 0.7114681 | 0.0000907 |
| C | 1.2544325 | 1.4014775 | −0.0000806 |
| C | 2.4469775 | 0.7298708 | −0.0002031 |
| C | 2.4358170 | −0.6943191 | −0.0003130 |
| C | 1.2418752 | −1.3517156 | 0.0000762 |
| N | 0.0202625 | −0.6961970 | 0.0004983 |
| H | −1.1020031 | −2.6688142 | 0.0023745 |
| H | −3.4874387 | −1.1680289 | −0.0000457 |
| H | −3.3599368 | 1.2958031 | −0.0009741 |
| H | −1.1954790 | 2.4715360 | −0.0003406 |
| H | 1.2214050 | 2.4913131 | −0.0001774 |
| H | 3.3922808 | 1.2729053 | −0.0002828 |
| H | 3.3617485 | −1.2671881 | −0.0006823 |
| H | 1.1646382 | −2.4374043 | −0.0000291 |

BN-9,2 naphthalene

| | | | |
|---|---|---|---|
| C | −1.1766925 | −1.4195316 | 0.0009420 |
| B | −2.5154824 | −0.7578508 | 0.0002817 |
| C | −2.4601298 | 0.7628032 | −0.0003879 |
| C | −1.2369382 | 1.3969272 | −0.0001751 |
| C | −0.0008100 | 0.7046154 | 0.0001709 |
| C | 1.2423733 | 1.3805793 | −0.0000346 |
| C | 2.4456958 | 0.7149396 | −0.0001763 |
| C | 2.4186994 | −0.7008093 | −0.0001958 |

| | | | |
|---|---|---|---|
| C | 1.2250323 | −1.3629278 | 0.0001893 |
| N | −0.0007329 | −0.7026917 | 0.0005285 |
| H | −0.9924036 | −2.4964490 | 0.0015524 |
| H | −3.5286933 | −1.4052242 | 0.0002432 |
| H | −3.3469270 | 1.4043055 | −0.0009350 |
| H | −1.1516841 | 2.4879293 | −0.0003982 |
| H | 1.2027442 | 2.4703171 | −0.0001890 |
| H | 3.3907657 | 1.2560345 | −0.0003580 |
| H | 3.3382663 | −1.2849654 | −0.0005601 |
| H | 1.1470167 | −2.4480014 | 0.0001020 |

BN-9,3 naphthalene

| | | | |
|---|---|---|---|
| C | −1.1951100 | −1.3735436 | 0.0008357 |
| C | −2.4220930 | −0.7807571 | 0.0003416 |
| B | −2.5422883 | 0.7431767 | −0.0004322 |
| C | −1.2158628 | 1.4522102 | −0.0001828 |
| C | −0.0096287 | 0.7599960 | 0.0001654 |
| C | 1.2773951 | 1.3944545 | 0.0000398 |
| C | 2.4493163 | 0.6932683 | −0.0001770 |
| C | 2.4028004 | −0.7318042 | −0.0002413 |
| C | 1.1945051 | −1.3560457 | 0.0001520 |
| N | −0.0036778 | −0.6571137 | 0.0004928 |
| H | −1.0378562 | −2.4543591 | 0.0014299 |
| H | −3.2716878 | −1.4691678 | 0.0005749 |
| H | −3.6010022 | 1.3149882 | −0.0011540 |
| H | −1.1045571 | 2.5404874 | −0.0003649 |
| H | 1.2732724 | 2.4844698 | −0.0000318 |
| H | 3.4087269 | 1.2108385 | −0.0003576 |
| H | 3.3109690 | −1.3317564 | −0.0006022 |
| H | 1.0868788 | −2.4393418 | 0.0001117 |

BN-9,10 naphthalene

| | | | |
|---|---|---|---|
| C | −1.3741926 | −1.4710494 | 0.0009804 |
| C | −2.5026068 | −0.6869119 | 0.0004059 |
| C | −2.4021366 | 0.7370426 | −0.0002800 |
| C | −1.1912760 | 1.3708952 | −0.0002482 |
| N | 0.0000089 | 0.6685611 | 0.0002084 |
| C | 1.1912822 | 1.3709049 | −0.0000095 |
| C | 2.4021646 | 0.7370190 | −0.0002471 |
| C | 2.5026291 | −0.6869057 | −0.0003098 |
| C | 1.3741857 | −1.4710700 | 0.0002324 |
| B | 0.0000027 | −0.8182877 | 0.0006051 |

| | | | |
|---|---|---|---|
| H | −1.4982086 | −2.5573905 | 0.0015263 |
| H | −3.5039934 | −1.1272923 | 0.0004602 |
| H | −3.3030782 | 1.3505835 | −0.0008245 |
| H | −1.1062616 | 2.4590198 | −0.0006396 |
| H | 1.1062823 | 2.4590092 | −0.0001192 |
| H | 3.3030941 | 1.3505706 | −0.0004582 |
| H | 3.5039963 | −1.1273070 | −0.0008045 |
| H | 1.4982079 | −2.5573915 | 0.0001219 |

BN-10,1 naphthalene

| | | | |
|---|---|---|---|
| B | −1.2979040 | −1.5203183 | 0.0010706 |
| C | −2.5378521 | −0.6815968 | 0.0003140 |
| C | −2.4049295 | 0.7121187 | −0.0003429 |
| C | −1.1762563 | 1.3527433 | −0.0002560 |
| N | 0.0110845 | 0.6601799 | 0.0001538 |
| C | 1.2052307 | 1.3711758 | −0.0000423 |
| C | 2.4169919 | 0.7435982 | −0.0002883 |
| C | 2.4822533 | −0.6714122 | −0.0003290 |
| C | 1.2968865 | −1.3731278 | 0.0002430 |
| C | 0.0244178 | −0.7522576 | 0.0006073 |
| H | −1.2627603 | −2.7237333 | 0.0018366 |
| H | −3.5548768 | −1.0831156 | 0.0001516 |
| H | −3.2808937 | 1.3675679 | −0.0009339 |
| H | −1.0762147 | 2.4361446 | −0.0005377 |
| H | 1.0970573 | 2.4542998 | −0.0001405 |
| H | 3.3198367 | 1.3534702 | −0.0004755 |
| H | 3.4446552 | −1.1821949 | −0.0007518 |
| H | 1.2933734 | −2.4635419 | 0.0003211 |

# S9 Partially relaxed geometries: benzene, naphthalene and its BN-doped derivatives

The structures of benzene and naphthalene were optimized at the CCSD level of theory. BN-doped variants were generated by replacing a pair of carbon atoms with nitrogen and boron without further re-optimization, and the atomic coordinates are presented in angstroms (Å).

benzene

| | | | |
|---|---|---|---|
| C | 0.1669048161 | −1.3960409438 | 0.0004259008 |
| C | −1.1255546043 | −0.8425643118 | 0.0005156376 |

| | | | |
|---|---|---|---|
| C | −1.2924594155 | 0.5534765466 | −0.0001304860 |
| C | −0.1669047614 | 1.3960405836 | −0.0005142176 |
| C | 1.1255545679 | 0.8425642659 | 0.0001738626 |
| C | 1.2924594506 | −0.5534767464 | 0.0002991077 |
| H | 0.2970044154 | −2.4842313184 | 0.0020909630 |
| H | −2.0029063788 | −1.4993294722 | 0.0007710594 |
| H | −2.2999107642 | 0.9849026577 | −0.0003209795 |
| H | −0.2970042864 | 2.4842319649 | −0.0012069892 |
| H | 2.0029063283 | 1.4993294350 | −0.0001962795 |
| H | 2.2999106322 | −0.9849026612 | 0.0010924208 |

1,2 azaborine

| | | | |
|---|---|---|---|
| N | 0.1669048161 | −1.3960409438 | 0.0004259008 |
| B | −1.1255546043 | −0.8425643118 | 0.0005156376 |
| C | −1.2924594155 | 0.5534765466 | −0.0001304860 |
| C | −0.1669047614 | 1.3960405836 | −0.0005142176 |
| C | 1.1255545679 | 0.8425642659 | 0.0001738626 |
| C | 1.2924594506 | −0.5534767464 | 0.0002991077 |
| H | 0.2970044154 | −2.4842313184 | 0.0020909630 |
| H | −2.0029063788 | −1.4993294722 | 0.0007710594 |
| H | −2.2999107642 | 0.9849026577 | −0.0003209795 |
| H | −0.2970042864 | 2.4842319649 | −0.0012069892 |
| H | 2.0029063283 | 1.4993294350 | −0.0001962795 |
| H | 2.2999106322 | −0.9849026612 | 0.0010924208 |

1,3 azaborine

| | | | |
|---|---|---|---|
| N | 0.1669048161 | −1.3960409438 | 0.0004259008 |
| C | −1.1255546043 | −0.8425643118 | 0.0005156376 |
| B | −1.2924594155 | 0.5534765466 | −0.0001304860 |
| C | −0.1669047614 | 1.3960405836 | −0.0005142176 |
| C | 1.1255545679 | 0.8425642659 | 0.0001738626 |
| C | 1.2924594506 | −0.5534767464 | 0.0002991077 |
| H | 0.2970044154 | −2.4842313184 | 0.0020909630 |
| H | −2.0029063788 | −1.4993294722 | 0.0007710594 |
| H | −2.2999107642 | 0.9849026577 | −0.0003209795 |
| H | −0.2970042864 | 2.4842319649 | −0.0012069892 |
| H | 2.0029063283 | 1.4993294350 | −0.0001962795 |
| H | 2.2999106322 | −0.9849026612 | 0.0010924208 |

1,4 azaborine

| | | | |
|---|---|---|---|
| N | 0.1669048161 | −1.3960409438 | 0.0004259008 |

| | | | |
|---|---|---|---|
| C | −1.1255546043 | −0.8425643118 | 0.0005156376 |
| C | −1.2924594155 | 0.5534765466 | −0.0001304860 |
| B | −0.1669047614 | 1.3960405836 | −0.0005142176 |
| C | 1.1255545679 | 0.8425642659 | 0.0001738626 |
| C | 1.2924594506 | −0.5534767464 | 0.0002991077 |
| H | 0.2970044154 | −2.4842313184 | 0.0020909630 |
| H | −2.0029063788 | −1.4993294722 | 0.0007710594 |
| H | −2.2999107642 | 0.9849026577 | −0.0003209795 |
| H | −0.2970042864 | 2.4842319649 | −0.0012069892 |
| H | 2.0029063283 | 1.4993294350 | −0.0001962795 |
| H | 2.2999106322 | −0.9849026612 | 0.0010924208 |

di-BN-doped benzene

| | | | |
|---|---|---|---|
| N | 0.1669048161 | −1.3960409438 | 0.0004259008 |
| B | −1.1255546043 | −0.8425643118 | 0.0005156376 |
| C | −1.2924594155 | 0.5534765466 | −0.0001304860 |
| N | −0.1669047614 | 1.3960405836 | −0.0005142176 |
| B | 1.1255545679 | 0.8425642659 | 0.0001738626 |
| C | 1.2924594506 | −0.5534767464 | 0.0002991077 |
| H | 0.2970044154 | −2.4842313184 | 0.0020909630 |
| H | −2.0029063788 | −1.4993294722 | 0.0007710594 |
| H | −2.2999107642 | 0.9849026577 | −0.0003209795 |
| H | −0.2970042864 | 2.4842319649 | −0.0012069892 |
| H | 2.0029063283 | 1.4993294350 | −0.0001962795 |
| H | 2.2999106322 | −0.9849026612 | 0.0010924208 |

Borazine

| | | | |
|---|---|---|---|
| N | 0.1669048161 | −1.3960409438 | 0.0004259008 |
| B | −1.1255546043 | −0.8425643118 | 0.0005156376 |
| N | −1.2924594155 | 0.5534765466 | −0.0001304860 |
| B | −0.1669047614 | 1.3960405836 | −0.0005142176 |
| N | 1.1255545679 | 0.8425642659 | 0.0001738626 |
| B | 1.2924594506 | −0.5534767464 | 0.0002991077 |
| H | 0.2970044154 | −2.4842313184 | 0.0020909630 |
| H | −2.0029063788 | −1.4993294722 | 0.0007710594 |
| H | −2.2999107642 | 0.9849026577 | −0.0003209795 |
| H | −0.2970042864 | 2.4842319649 | −0.0012069892 |
| H | 2.0029063283 | 1.4993294350 | −0.0001962795 |
| H | 2.2999106322 | −0.9849026612 | 0.0010924208 |

naphthalene

| | | | |
|---|---|---|---|
| C | −1.2535277763 | −1.4110794238 | −0.0002197172 |
| C | −2.4487573574 | −0.7151974344 | 0.0004030475 |
| C | −2.4487543911 | 0.7152070489 | 0.0000134416 |
| C | −1.2535219120 | 1.4110840670 | −0.0002294522 |
| C | 0.0000072165 | 0.7147110586 | −0.0000953346 |
| C | 1.2535390831 | 1.4110792209 | −0.0002622781 |
| C | 2.4487686680 | 0.7151972371 | −0.0001528388 |
| C | 2.4487653922 | −0.7152070555 | 0.0001706221 |
| C | 1.2535328716 | −1.4110840936 | 0.0002628364 |
| C | 0.0000040322 | −0.7147108268 | 0.0000946476 |
| H | −1.2485032556 | −2.5076954610 | 0.0012065053 |
| H | −3.4011845526 | −1.2570132418 | 0.0007843569 |
| H | −3.4011793763 | 1.2570267297 | −0.0000288633 |
| H | −1.2484932950 | 2.5077008461 | −0.0005950142 |
| H | 1.2485149429 | 2.5076960454 | −0.0006027638 |
| H | 3.4011956518 | 1.2570128442 | −0.0004592223 |
| H | 3.4011900882 | −1.2570266855 | 0.0001599108 |
| H | 1.2485039699 | −2.5077008755 | 0.0001501165 |

BN-1,2 naphthalene

| | | | |
|---|---|---|---|
| N | −1.2535277763 | −1.4110794238 | −0.0002197172 |
| B | −2.4487573574 | −0.7151974344 | 0.0004030475 |
| C | −2.4487543911 | 0.7152070489 | 0.0000134416 |
| C | −1.2535219120 | 1.4110840670 | −0.0002294522 |
| C | 0.0000072165 | 0.7147110586 | −0.0000953346 |
| C | 1.2535390831 | 1.4110792209 | −0.0002622781 |
| C | 2.4487686680 | 0.7151972371 | −0.0001528388 |
| C | 2.4487653922 | −0.7152070555 | 0.0001706221 |
| C | 1.2535328716 | −1.4110840936 | 0.0002628364 |
| C | 0.0000040322 | −0.7147108268 | 0.0000946476 |
| H | −1.2485032556 | −2.5076954610 | 0.0012065053 |
| H | −3.4011845526 | −1.2570132418 | 0.0007843569 |
| H | −3.4011793763 | 1.2570267297 | −0.0000288633 |
| H | −1.2484932950 | 2.5077008461 | −0.0005950142 |
| H | 1.2485149429 | 2.5076960454 | −0.0006027638 |
| H | 3.4011956518 | 1.2570128442 | −0.0004592223 |
| H | 3.4011900882 | −1.2570266855 | 0.0001599108 |
| H | 1.2485039699 | −2.5077008755 | 0.0001501165 |

BN-1,3 naphthalene

| | | | |
|---|---|---|---|
| N | −1.2535277763 | −1.4110794238 | −0.0002197172 |
| C | −2.4487573574 | −0.7151974344 | 0.0004030475 |

| | | | |
|---|---|---|---|
| B | −2.4487543911 | 0.7152070489 | 0.0000134416 |
| C | −1.2535219120 | 1.4110840670 | −0.0002294522 |
| C | 0.0000072165 | 0.7147110586 | −0.0000953346 |
| C | 1.2535390831 | 1.4110792209 | −0.0002622781 |
| C | 2.4487686680 | 0.7151972371 | −0.0001528388 |
| C | 2.4487653922 | −0.7152070555 | 0.0001706221 |
| C | 1.2535328716 | −1.4110840936 | 0.0002628364 |
| C | 0.0000040322 | −0.7147108268 | 0.0000946476 |
| H | −1.2485032556 | −2.5076954610 | 0.0012065053 |
| H | −3.4011845526 | −1.2570132418 | 0.0007843569 |
| H | −3.4011793763 | 1.2570267297 | −0.0000288633 |
| H | −1.2484932950 | 2.5077008461 | −0.0005950142 |
| H | 1.2485149429 | 2.5076960454 | −0.0006027638 |
| H | 3.4011956518 | 1.2570128442 | −0.0004592223 |
| H | 3.4011900882 | −1.2570266855 | 0.0001599108 |
| H | 1.2485039699 | −2.5077008755 | 0.0001501165 |

BN-1,4 naphthalene

| | | | |
|---|---|---|---|
| N | −1.2535277763 | −1.4110794238 | −0.0002197172 |
| C | −2.4487573574 | −0.7151974344 | 0.0004030475 |
| C | −2.4487543911 | 0.7152070489 | 0.0000134416 |
| B | −1.2535219120 | 1.4110840670 | −0.0002294522 |
| C | 0.0000072165 | 0.7147110586 | −0.0000953346 |
| C | 1.2535390831 | 1.4110792209 | −0.0002622781 |
| C | 2.4487686680 | 0.7151972371 | −0.0001528388 |
| C | 2.4487653922 | −0.7152070555 | 0.0001706221 |
| C | 1.2535328716 | −1.4110840936 | 0.0002628364 |
| C | 0.0000040322 | −0.7147108268 | 0.0000946476 |
| H | −1.2485032556 | −2.5076954610 | 0.0012065053 |
| H | −3.4011845526 | −1.2570132418 | 0.0007843569 |
| H | −3.4011793763 | 1.2570267297 | −0.0000288633 |
| H | −1.2484932950 | 2.5077008461 | −0.0005950142 |
| H | 1.2485149429 | 2.5076960454 | −0.0006027638 |
| H | 3.4011956518 | 1.2570128442 | −0.0004592223 |
| H | 3.4011900882 | −1.2570266855 | 0.0001599108 |
| H | 1.2485039699 | −2.5077008755 | 0.0001501165 |

BN-1,5 naphthalene

| | | | |
|---|---|---|---|
| N | −1.2535277763 | −1.4110794238 | −0.0002197172 |
| C | −2.4487573574 | −0.7151974344 | 0.0004030475 |
| C | −2.4487543911 | 0.7152070489 | 0.0000134416 |
| C | −1.2535219120 | 1.4110840670 | −0.0002294522 |

| | | | |
|---|---|---|---|
| C | 0.0000072165 | 0.7147110586 | −0.0000953346 |
| B | 1.2535390831 | 1.4110792209 | −0.0002622781 |
| C | 2.4487686680 | 0.7151972371 | −0.0001528388 |
| C | 2.4487653922 | −0.7152070555 | 0.0001706221 |
| C | 1.2535328716 | −1.4110840936 | 0.0002628364 |
| C | 0.0000040322 | −0.7147108268 | 0.0000946476 |
| H | −1.2485032556 | −2.5076954610 | 0.0012065053 |
| H | −3.4011845526 | −1.2570132418 | 0.0007843569 |
| H | −3.4011793763 | 1.2570267297 | −0.0000288633 |
| H | −1.2484932950 | 2.5077008461 | −0.0005950142 |
| H | 1.2485149429 | 2.5076960454 | −0.0006027638 |
| H | 3.4011956518 | 1.2570128442 | −0.0004592223 |
| H | 3.4011900882 | −1.2570266855 | 0.0001599108 |
| H | 1.2485039699 | −2.5077008755 | 0.0001501165 |

BN-1,6 naphthalene

| | | | |
|---|---|---|---|
| N | −1.2535277763 | −1.4110794238 | −0.0002197172 |
| C | −2.4487573574 | −0.7151974344 | 0.0004030475 |
| C | −2.4487543911 | 0.7152070489 | 0.0000134416 |
| C | −1.2535219120 | 1.4110840670 | −0.0002294522 |
| C | 0.0000072165 | 0.7147110586 | −0.0000953346 |
| C | 1.2535390831 | 1.4110792209 | −0.0002622781 |
| B | 2.4487686680 | 0.7151972371 | −0.0001528388 |
| C | 2.4487653922 | −0.7152070555 | 0.0001706221 |
| C | 1.2535328716 | −1.4110840936 | 0.0002628364 |
| C | 0.0000040322 | −0.7147108268 | 0.0000946476 |
| H | −1.2485032556 | −2.5076954610 | 0.0012065053 |
| H | −3.4011845526 | −1.2570132418 | 0.0007843569 |
| H | −3.4011793763 | 1.2570267297 | −0.0000288633 |
| H | −1.2484932950 | 2.5077008461 | −0.0005950142 |
| H | 1.2485149429 | 2.5076960454 | −0.0006027638 |
| H | 3.4011956518 | 1.2570128442 | −0.0004592223 |
| H | 3.4011900882 | −1.2570266855 | 0.0001599108 |
| H | 1.2485039699 | −2.5077008755 | 0.0001501165 |

BN-1,7 naphthalene

| | | | |
|---|---|---|---|
| N | −1.2535277763 | −1.4110794238 | −0.0002197172 |
| C | −2.4487573574 | −0.7151974344 | 0.0004030475 |
| C | −2.4487543911 | 0.7152070489 | 0.0000134416 |
| C | −1.2535219120 | 1.4110840670 | −0.0002294522 |
| C | 0.0000072165 | 0.7147110586 | −0.0000953346 |
| C | 1.2535390831 | 1.4110792209 | −0.0002622781 |

| | | | |
|---|---|---|---|
| C | 2.4487686680 | 0.7151972371 | −0.0001528388 |
| B | 2.4487653922 | −0.7152070555 | 0.0001706221 |
| C | 1.2535328716 | −1.4110840936 | 0.0002628364 |
| C | 0.0000040322 | −0.7147108268 | 0.0000946476 |
| H | −1.2485032556 | −2.5076954610 | 0.0012065053 |
| H | −3.4011845526 | −1.2570132418 | 0.0007843569 |
| H | −3.4011793763 | 1.2570267297 | −0.0000288633 |
| H | −1.2484932950 | 2.5077008461 | −0.0005950142 |
| H | 1.2485149429 | 2.5076960454 | −0.0006027638 |
| H | 3.4011956518 | 1.2570128442 | −0.0004592223 |
| H | 3.4011900882 | −1.2570266855 | 0.0001599108 |
| H | 1.2485039699 | −2.5077008755 | 0.0001501165 |

BN-1,8 naphthalene

| | | | |
|---|---|---|---|
| N | −1.2535277763 | −1.4110794238 | −0.0002197172 |
| C | −2.4487573574 | −0.7151974344 | 0.0004030475 |
| C | −2.4487543911 | 0.7152070489 | 0.0000134416 |
| C | −1.2535219120 | 1.4110840670 | −0.0002294522 |
| C | 0.0000072165 | 0.7147110586 | −0.0000953346 |
| C | 1.2535390831 | 1.4110792209 | −0.0002622781 |
| C | 2.4487686680 | 0.7151972371 | −0.0001528388 |
| C | 2.4487653922 | −0.7152070555 | 0.0001706221 |
| B | 1.2535328716 | −1.4110840936 | 0.0002628364 |
| C | 0.0000040322 | −0.7147108268 | 0.0000946476 |
| H | −1.2485032556 | −2.5076954610 | 0.0012065053 |
| H | −3.4011845526 | −1.2570132418 | 0.0007843569 |
| H | −3.4011793763 | 1.2570267297 | −0.0000288633 |
| H | −1.2484932950 | 2.5077008461 | −0.0005950142 |
| H | 1.2485149429 | 2.5076960454 | −0.0006027638 |
| H | 3.4011956518 | 1.2570128442 | −0.0004592223 |
| H | 3.4011900882 | −1.2570266855 | 0.0001599108 |
| H | 1.2485039699 | −2.5077008755 | 0.0001501165 |

BN-1,9 naphthalene

| | | | |
|---|---|---|---|
| N | −1.2535277763 | −1.4110794238 | −0.0002197172 |
| C | −2.4487573574 | −0.7151974344 | 0.0004030475 |
| C | −2.4487543911 | 0.7152070489 | 0.0000134416 |
| C | −1.2535219120 | 1.4110840670 | −0.0002294522 |
| C | 0.0000072165 | 0.7147110586 | −0.0000953346 |
| C | 1.2535390831 | 1.4110792209 | −0.0002622781 |
| C | 2.4487686680 | 0.7151972371 | −0.0001528388 |
| C | 2.4487653922 | −0.7152070555 | 0.0001706221 |

| | | | |
|---|---|---|---|
| C | 1.2535328716 | −1.4110840936 | 0.0002628364 |
| B | 0.0000040322 | −0.7147108268 | 0.0000946476 |
| H | −1.2485032556 | −2.5076954610 | 0.0012065053 |
| H | −3.4011845526 | −1.2570132418 | 0.0007843569 |
| H | −3.4011793763 | 1.2570267297 | −0.0000288633 |
| H | −1.2484932950 | 2.5077008461 | −0.0005950142 |
| H | 1.2485149429 | 2.5076960454 | −0.0006027638 |
| H | 3.4011956518 | 1.2570128442 | −0.0004592223 |
| H | 3.4011900882 | −1.2570266855 | 0.0001599108 |
| H | 1.2485039699 | −2.5077008755 | 0.0001501165 |

BN-1,10 naphthalene

| | | | |
|---|---|---|---|
| N | −1.2535277763 | −1.4110794238 | −0.0002197172 |
| C | −2.4487573574 | −0.7151974344 | 0.0004030475 |
| C | −2.4487543911 | 0.7152070489 | 0.0000134416 |
| C | −1.2535219120 | 1.4110840670 | −0.0002294522 |
| B | 0.0000072165 | 0.7147110586 | −0.0000953346 |
| C | 1.2535390831 | 1.4110792209 | −0.0002622781 |
| C | 2.4487686680 | 0.7151972371 | −0.0001528388 |
| C | 2.4487653922 | −0.7152070555 | 0.0001706221 |
| C | 1.2535328716 | −1.4110840936 | 0.0002628364 |
| C | 0.0000040322 | −0.7147108268 | 0.0000946476 |
| H | −1.2485032556 | −2.5076954610 | 0.0012065053 |
| H | −3.4011845526 | −1.2570132418 | 0.0007843569 |
| H | −3.4011793763 | 1.2570267297 | −0.0000288633 |
| H | −1.2484932950 | 2.5077008461 | −0.0005950142 |
| H | 1.2485149429 | 2.5076960454 | −0.0006027638 |
| H | 3.4011956518 | 1.2570128442 | −0.0004592223 |
| H | 3.4011900882 | −1.2570266855 | 0.0001599108 |
| H | 1.2485039699 | −2.5077008755 | 0.0001501165 |

BN-2,1 naphthalene

| | | | |
|---|---|---|---|
| B | −1.2535277763 | −1.4110794238 | −0.0002197172 |
| N | −2.4487573574 | −0.7151974344 | 0.0004030475 |
| C | −2.4487543911 | 0.7152070489 | 0.0000134416 |
| C | −1.2535219120 | 1.4110840670 | −0.0002294522 |
| C | 0.0000072165 | 0.7147110586 | −0.0000953346 |
| C | 1.2535390831 | 1.4110792209 | −0.0002622781 |
| C | 2.4487686680 | 0.7151972371 | −0.0001528388 |
| C | 2.4487653922 | −0.7152070555 | 0.0001706221 |
| C | 1.2535328716 | −1.4110840936 | 0.0002628364 |
| C | 0.0000040322 | −0.7147108268 | 0.0000946476 |

| | | | |
|---|---|---|---|
| H | −1.2485032556 | −2.5076954610 | 0.0012065053 |
| H | −3.4011845526 | −1.2570132418 | 0.0007843569 |
| H | −3.4011793763 | 1.2570267297 | −0.0000288633 |
| H | −1.2484932950 | 2.5077008461 | −0.0005950142 |
| H | 1.2485149429 | 2.5076960454 | −0.0006027638 |
| H | 3.4011956518 | 1.2570128442 | −0.0004592223 |
| H | 3.4011900882 | −1.2570266855 | 0.0001599108 |
| H | 1.2485039699 | −2.5077008755 | 0.0001501165 |

BN-2,3 naphthalene

| | | | |
|---|---|---|---|
| C | −1.2535277763 | −1.4110794238 | −0.0002197172 |
| N | −2.4487573574 | −0.7151974344 | 0.0004030475 |
| B | −2.4487543911 | 0.7152070489 | 0.0000134416 |
| C | −1.2535219120 | 1.4110840670 | −0.0002294522 |
| C | 0.0000072165 | 0.7147110586 | −0.0000953346 |
| C | 1.2535390831 | 1.4110792209 | −0.0002622781 |
| C | 2.4487686680 | 0.7151972371 | −0.0001528388 |
| C | 2.4487653922 | −0.7152070555 | 0.0001706221 |
| C | 1.2535328716 | −1.4110840936 | 0.0002628364 |
| C | 0.0000040322 | −0.7147108268 | 0.0000946476 |
| H | −1.2485032556 | −2.5076954610 | 0.0012065053 |
| H | −3.4011845526 | −1.2570132418 | 0.0007843569 |
| H | −3.4011793763 | 1.2570267297 | −0.0000288633 |
| H | −1.2484932950 | 2.5077008461 | −0.0005950142 |
| H | 1.2485149429 | 2.5076960454 | −0.0006027638 |
| H | 3.4011956518 | 1.2570128442 | −0.0004592223 |
| H | 3.4011900882 | −1.2570266855 | 0.0001599108 |
| H | 1.2485039699 | −2.5077008755 | 0.0001501165 |

BN-2,4 naphthalene

| | | | |
|---|---|---|---|
| C | −1.2535277763 | −1.4110794238 | −0.0002197172 |
| N | −2.4487573574 | −0.7151974344 | 0.0004030475 |
| C | −2.4487543911 | 0.7152070489 | 0.0000134416 |
| B | −1.2535219120 | 1.4110840670 | −0.0002294522 |
| C | 0.0000072165 | 0.7147110586 | −0.0000953346 |
| C | 1.2535390831 | 1.4110792209 | −0.0002622781 |
| C | 2.4487686680 | 0.7151972371 | −0.0001528388 |
| C | 2.4487653922 | −0.7152070555 | 0.0001706221 |
| C | 1.2535328716 | −1.4110840936 | 0.0002628364 |
| C | 0.0000040322 | −0.7147108268 | 0.0000946476 |
| H | −1.2485032556 | −2.5076954610 | 0.0012065053 |
| H | −3.4011845526 | −1.2570132418 | 0.0007843569 |

| | | | |
|---|---|---|---|
| H | −3.4011793763 | 1.2570267297 | −0.0000288633 |
| H | −1.2484932950 | 2.5077008461 | −0.0005950142 |
| H | 1.2485149429 | 2.5076960454 | −0.0006027638 |
| H | 3.4011956518 | 1.2570128442 | −0.0004592223 |
| H | 3.4011900882 | −1.2570266855 | 0.0001599108 |
| H | 1.2485039699 | −2.5077008755 | 0.0001501165 |

BN-2,5 naphthalene

| | | | |
|---|---|---|---|
| C | −1.2535277763 | −1.4110794238 | −0.0002197172 |
| N | −2.4487573574 | −0.7151974344 | 0.0004030475 |
| C | −2.4487543911 | 0.7152070489 | 0.0000134416 |
| C | −1.2535219120 | 1.4110840670 | −0.0002294522 |
| C | 0.0000072165 | 0.7147110586 | −0.0000953346 |
| B | 1.2535390831 | 1.4110792209 | −0.0002622781 |
| C | 2.4487686680 | 0.7151972371 | −0.0001528388 |
| C | 2.4487653922 | −0.7152070555 | 0.0001706221 |
| C | 1.2535328716 | −1.4110840936 | 0.0002628364 |
| C | 0.0000040322 | −0.7147108268 | 0.0000946476 |
| H | −1.2485032556 | −2.5076954610 | 0.0012065053 |
| H | −3.4011845526 | −1.2570132418 | 0.0007843569 |
| H | −3.4011793763 | 1.2570267297 | −0.0000288633 |
| H | −1.2484932950 | 2.5077008461 | −0.0005950142 |
| H | 1.2485149429 | 2.5076960454 | −0.0006027638 |
| H | 3.4011956518 | 1.2570128442 | −0.0004592223 |
| H | 3.4011900882 | −1.2570266855 | 0.0001599108 |
| H | 1.2485039699 | −2.5077008755 | 0.000150116 |

BN-2,6 naphthalene

| | | | |
|---|---|---|---|
| C | −1.2535277763 | −1.4110794238 | −0.0002197172 |
| N | −2.4487573574 | −0.7151974344 | 0.0004030475 |
| C | −2.4487543911 | 0.7152070489 | 0.0000134416 |
| C | −1.2535219120 | 1.4110840670 | −0.0002294522 |
| C | 0.0000072165 | 0.7147110586 | −0.0000953346 |
| C | 1.2535390831 | 1.4110792209 | −0.0002622781 |
| B | 2.4487686680 | 0.7151972371 | −0.0001528388 |
| C | 2.4487653922 | −0.7152070555 | 0.0001706221 |
| C | 1.2535328716 | −1.4110840936 | 0.0002628364 |
| C | 0.0000040322 | −0.7147108268 | 0.0000946476 |
| H | −1.2485032556 | −2.5076954610 | 0.0012065053 |
| H | −3.4011845526 | −1.2570132418 | 0.0007843569 |
| H | −3.4011793763 | 1.2570267297 | −0.0000288633 |
| H | −1.2484932950 | 2.5077008461 | −0.0005950142 |

| | | | |
|---|---|---|---|
| H | 1.2485149429 | 2.5076960454 | −0.0006027638 |
| H | 3.4011956518 | 1.2570128442 | −0.0004592223 |
| H | 3.4011900882 | −1.2570266855 | 0.0001599108 |
| H | 1.2485039699 | −2.5077008755 | 0.0001501165 |

BN-2,7 naphthalene

| | | | |
|---|---|---|---|
| C | −1.2535277763 | −1.4110794238 | −0.0002197172 |
| N | −2.4487573574 | −0.7151974344 | 0.0004030475 |
| C | −2.4487543911 | 0.7152070489 | 0.0000134416 |
| C | −1.2535219120 | 1.4110840670 | −0.0002294522 |
| C | 0.0000072165 | 0.7147110586 | −0.0000953346 |
| C | 1.2535390831 | 1.4110792209 | −0.0002622781 |
| C | 2.4487686680 | 0.7151972371 | −0.0001528388 |
| B | 2.4487653922 | −0.7152070555 | 0.0001706221 |
| C | 1.2535328716 | −1.4110840936 | 0.0002628364 |
| C | 0.0000040322 | −0.7147108268 | 0.0000946476 |
| H | −1.2485032556 | −2.5076954610 | 0.0012065053 |
| H | −3.4011845526 | −1.2570132418 | 0.0007843569 |
| H | −3.4011793763 | 1.2570267297 | −0.0000288633 |
| H | −1.2484932950 | 2.5077008461 | −0.0005950142 |
| H | 1.2485149429 | 2.5076960454 | −0.0006027638 |
| H | 3.4011956518 | 1.2570128442 | −0.0004592223 |
| H | 3.4011900882 | −1.2570266855 | 0.0001599108 |
| H | 1.2485039699 | −2.5077008755 | 0.0001501165 |

BN-2,8 naphthalene

| | | | |
|---|---|---|---|
| C | −1.2535277763 | −1.4110794238 | −0.0002197172 |
| N | −2.4487573574 | −0.7151974344 | 0.0004030475 |
| C | −2.4487543911 | 0.7152070489 | 0.0000134416 |
| C | −1.2535219120 | 1.4110840670 | −0.0002294522 |
| C | 0.0000072165 | 0.7147110586 | −0.0000953346 |
| C | 1.2535390831 | 1.4110792209 | −0.0002622781 |
| C | 2.4487686680 | 0.7151972371 | −0.0001528388 |
| C | 2.4487653922 | −0.7152070555 | 0.0001706221 |
| B | 1.2535328716 | −1.4110840936 | 0.0002628364 |
| C | 0.0000040322 | −0.7147108268 | 0.0000946476 |
| H | −1.2485032556 | −2.5076954610 | 0.0012065053 |
| H | −3.4011845526 | −1.2570132418 | 0.0007843569 |
| H | −3.4011793763 | 1.2570267297 | −0.0000288633 |
| H | −1.2484932950 | 2.5077008461 | −0.0005950142 |
| H | 1.2485149429 | 2.5076960454 | −0.0006027638 |
| H | 3.4011956518 | 1.2570128442 | −0.0004592223 |

| | | | |
|---|---|---|---|
| H | 3.4011900882 | −1.2570266855 | 0.0001599108 |
| H | 1.2485039699 | −2.5077008755 | 0.0001501165 |

BN-2,9 naphthalene

| | | | |
|---|---|---|---|
| C | −1.2535277763 | −1.4110794238 | −0.0002197172 |
| N | −2.4487573574 | −0.7151974344 | 0.0004030475 |
| C | −2.4487543911 | 0.7152070489 | 0.0000134416 |
| C | −1.2535219120 | 1.4110840670 | −0.0002294522 |
| C | 0.0000072165 | 0.7147110586 | −0.0000953346 |
| C | 1.2535390831 | 1.4110792209 | −0.0002622781 |
| C | 2.4487686680 | 0.7151972371 | −0.0001528388 |
| C | 2.4487653922 | −0.7152070555 | 0.0001706221 |
| C | 1.2535328716 | −1.4110840936 | 0.0002628364 |
| B | 0.0000040322 | −0.7147108268 | 0.0000946476 |
| H | −1.2485032556 | −2.5076954610 | 0.0012065053 |
| H | −3.4011845526 | −1.2570132418 | 0.0007843569 |
| H | −3.4011793763 | 1.2570267297 | −0.0000288633 |
| H | −1.2484932950 | 2.5077008461 | −0.0005950142 |
| H | 1.2485149429 | 2.5076960454 | −0.0006027638 |
| H | 3.4011956518 | 1.2570128442 | −0.0004592223 |
| H | 3.4011900882 | −1.2570266855 | 0.0001599108 |
| H | 1.2485039699 | −2.5077008755 | 0.0001501165 |

BN-3,9 naphthalene

| | | | |
|---|---|---|---|
| C | −1.2535277763 | −1.4110794238 | −0.0002197172 |
| C | −2.4487573574 | −0.7151974344 | 0.0004030475 |
| N | −2.4487543911 | 0.7152070489 | 0.0000134416 |
| C | −1.2535219120 | 1.4110840670 | −0.0002294522 |
| C | 0.0000072165 | 0.7147110586 | −0.0000953346 |
| C | 1.2535390831 | 1.4110792209 | −0.0002622781 |
| C | 2.4487686680 | 0.7151972371 | −0.0001528388 |
| C | 2.4487653922 | −0.7152070555 | 0.0001706221 |
| C | 1.2535328716 | −1.4110840936 | 0.0002628364 |
| B | 0.0000040322 | −0.7147108268 | 0.0000946476 |
| H | −1.2485032556 | −2.5076954610 | 0.0012065053 |
| H | −3.4011845526 | −1.2570132418 | 0.0007843569 |
| H | −3.4011793763 | 1.2570267297 | −0.0000288633 |
| H | −1.2484932950 | 2.5077008461 | −0.0005950142 |
| H | 1.2485149429 | 2.5076960454 | −0.0006027638 |
| H | 3.4011956518 | 1.2570128442 | −0.0004592223 |
| H | 3.4011900882 | −1.2570266855 | 0.0001599108 |
| H | 1.2485039699 | −2.5077008755 | 0.0001501165 |

BN-9,1 naphthalene

| | | | |
|---|---|---|---|
| B | −1.2535277763 | −1.4110794238 | −0.0002197172 |
| C | −2.4487573574 | −0.7151974344 | 0.0004030475 |
| C | −2.4487543911 | 0.7152070489 | 0.0000134416 |
| C | −1.2535219120 | 1.4110840670 | −0.0002294522 |
| C | 0.0000072165 | 0.7147110586 | −0.0000953346 |
| C | 1.2535390831 | 1.4110792209 | −0.0002622781 |
| C | 2.4487686680 | 0.7151972371 | −0.0001528388 |
| C | 2.4487653922 | −0.7152070555 | 0.0001706221 |
| C | 1.2535328716 | −1.4110840936 | 0.0002628364 |
| N | 0.0000040322 | −0.7147108268 | 0.0000946476 |
| H | −1.2485032556 | −2.5076954610 | 0.0012065053 |
| H | −3.4011845526 | −1.2570132418 | 0.0007843569 |
| H | −3.4011793763 | 1.2570267297 | −0.0000288633 |
| H | −1.2484932950 | 2.5077008461 | −0.0005950142 |
| H | 1.2485149429 | 2.5076960454 | −0.0006027638 |
| H | 3.4011956518 | 1.2570128442 | −0.0004592223 |
| H | 3.4011900882 | −1.2570266855 | 0.0001599108 |
| H | 1.2485039699 | −2.5077008755 | 0.0001501165 |

BN-9,2 naphthalene

| | | | |
|---|---|---|---|
| C | −1.2535277763 | −1.4110794238 | −0.0002197172 |
| B | −2.4487573574 | −0.7151974344 | 0.0004030475 |
| C | −2.4487543911 | 0.7152070489 | 0.0000134416 |
| C | −1.2535219120 | 1.4110840670 | −0.0002294522 |
| C | 0.0000072165 | 0.7147110586 | −0.0000953346 |
| C | 1.2535390831 | 1.4110792209 | −0.0002622781 |
| C | 2.4487686680 | 0.7151972371 | −0.0001528388 |
| C | 2.4487653922 | −0.7152070555 | 0.0001706221 |
| C | 1.2535328716 | −1.4110840936 | 0.0002628364 |
| N | 0.0000040322 | −0.7147108268 | 0.0000946476 |
| H | −1.2485032556 | −2.5076954610 | 0.0012065053 |
| H | −3.4011845526 | −1.2570132418 | 0.0007843569 |
| H | −3.4011793763 | 1.2570267297 | −0.0000288633 |
| H | −1.2484932950 | 2.5077008461 | −0.0005950142 |
| H | 1.2485149429 | 2.5076960454 | −0.0006027638 |
| H | 3.4011956518 | 1.2570128442 | −0.0004592223 |
| H | 3.4011900882 | −1.2570266855 | 0.0001599108 |
| H | 1.2485039699 | −2.5077008755 | 0.0001501165 |

BN-9,3 naphthalene

| | | | |
|---|---|---|---|
| C | −1.2535277763 | −1.4110794238 | −0.0002197172 |
| C | −2.4487573574 | −0.7151974344 | 0.0004030475 |
| B | −2.4487543911 | 0.7152070489 | 0.0000134416 |
| C | −1.2535219120 | 1.4110840670 | −0.0002294522 |
| C | 0.0000072165 | 0.7147110586 | −0.0000953346 |
| C | 1.2535390831 | 1.4110792209 | −0.0002622781 |
| C | 2.4487686680 | 0.7151972371 | −0.0001528388 |
| C | 2.4487653922 | −0.7152070555 | 0.0001706221 |
| C | 1.2535328716 | −1.4110840936 | 0.0002628364 |
| N | 0.0000040322 | −0.7147108268 | 0.0000946476 |
| H | −1.2485032556 | −2.5076954610 | 0.0012065053 |
| H | −3.4011845526 | −1.2570132418 | 0.0007843569 |
| H | −3.4011793763 | 1.2570267297 | −0.0000288633 |
| H | −1.2484932950 | 2.5077008461 | −0.0005950142 |
| H | 1.2485149429 | 2.5076960454 | −0.0006027638 |
| H | 3.4011956518 | 1.2570128442 | −0.0004592223 |
| H | 3.4011900882 | −1.2570266855 | 0.0001599108 |
| H | 1.2485039699 | −2.5077008755 | 0.0001501165 |

BN-9,10 naphthalene

| | | | |
|---|---|---|---|
| C | −1.2535277763 | −1.4110794238 | −0.0002197172 |
| C | −2.4487573574 | −0.7151974344 | 0.0004030475 |
| C | −2.4487543911 | 0.7152070489 | 0.0000134416 |
| C | −1.2535219120 | 1.4110840670 | −0.0002294522 |
| N | 0.0000072165 | 0.7147110586 | −0.0000953346 |
| C | 1.2535390831 | 1.4110792209 | −0.0002622781 |
| C | 2.4487686680 | 0.7151972371 | −0.0001528388 |
| C | 2.4487653922 | −0.7152070555 | 0.0001706221 |
| C | 1.2535328716 | −1.4110840936 | 0.0002628364 |
| B | 0.0000040322 | −0.7147108268 | 0.0000946476 |
| H | −1.2485032556 | −2.5076954610 | 0.0012065053 |
| H | −3.4011845526 | −1.2570132418 | 0.0007843569 |
| H | −3.4011793763 | 1.2570267297 | −0.0000288633 |
| H | −1.2484932950 | 2.5077008461 | −0.0005950142 |
| H | 1.2485149429 | 2.5076960454 | −0.0006027638 |
| H | 3.4011956518 | 1.2570128442 | −0.0004592223 |
| H | 3.4011900882 | −1.2570266855 | 0.0001599108 |
| H | 1.2485039699 | −2.5077008755 | 0.0001501165 |

BN-10,1 naphthalene

| | | | |
|---|---|---|---|
| B | −1.2535277763 | −1.4110794238 | −0.0002197172 |
| C | −2.4487573574 | −0.7151974344 | 0.0004030475 |

| | | | |
|---|---|---|---|
| C | −2.4487543911 | 0.7152070489 | 0.0000134416 |
| C | −1.2535219120 | 1.4110840670 | −0.0002294522 |
| N | 0.0000072165 | 0.7147110586 | −0.0000953346 |
| C | 1.2535390831 | 1.4110792209 | −0.0002622781 |
| C | 2.4487686680 | 0.7151972371 | −0.0001528388 |
| C | 2.4487653922 | −0.7152070555 | 0.0001706221 |
| C | 1.2535328716 | −1.4110840936 | 0.0002628364 |
| C | 0.0000040322 | −0.7147108268 | 0.0000946476 |
| H | −1.2485032556 | −2.5076954610 | 0.0012065053 |
| H | −3.4011845526 | −1.2570132418 | 0.0007843569 |
| H | −3.4011793763 | 1.2570267297 | −0.0000288633 |
| H | −1.2484932950 | 2.5077008461 | −0.0005950142 |
| H | 1.2485149429 | 2.5076960454 | −0.0006027638 |
| H | 3.4011956518 | 1.2570128442 | −0.0004592223 |
| H | 3.4011900882 | −1.2570266855 | 0.0001599108 |
| H | 1.2485039699 | −2.5077008755 | 0.0001501165 |

# References


[1] A. Perera, R. W. Molt, V. F. Lotrich and R. J. Bartlett, in *Isaiah Shavitt: A Memorial Festschrift from Theoretical Chemistry Accounts*, ed. R. Shepard, R. M. Pitzer and T. Dunning, Springer Berlin Heidelberg, Berlin, Heidelberg, 2016, pp. 153–165.

[2] S. Gulania, E. F. Kjønstad, J. F. Stanton, H. Koch and A. I. Krylov, *J. Chem. Phys.*, 2021, **154**, 114115.

[3] M. Ravi, A. Perera, Y. C. Park and R. J. Bartlett, *J. Chem. Phys.*, 2023, **159**, 094101.

[4] C. J. Cramer, J. J. Nash and R. R. Squires, *Chem. Phys. Lett.*, 1997, **277**, 311–320.

[5] P. U. Manohar and A. I. Krylov, *J. Chem. Phys.*, 2008, **129**, 194105.

[6] L. V. Slipchenko and A. I. Krylov, *J. Chem. Phys.*, 2002, **117**, 4694–4708.

[7] P. G. Wenthold, R. R. Squires and W. C. Lineberger, *J. Am. Chem. Soc.*, 1998, **120**, 5279–5290.

[8] P. G. Wenthold, J. Hu and R. R. Squires, *J. Am. Chem. Soc.*, 1996, **118**, 11865–11871.

[9] L. V. Slipchenko and A. I. Krylov, *J. Chem. Phys.*, 2002, **117**, 4694–4708.

[10] J. O. Howell, J. M. Goncalves, C. Amatore, L. Klasinc, R. M. Wightman and J. K. Kochi, *J. Am. Chem. Soc.*, 1984, **106**, 3968–3976.

[11] A. Chrostowska, S. Xu, A. N. Lamm, A. Mazière, C. D. Weber, A. Dargelos, P. Baylère, A. Graciaa and S.-Y. Liu, *J. Am. Chem. Soc.*, 2012, **134**, 10279–10285.

[12] C. R. Brundle, M. B. Robin and N. A. Kuebler, *J. Am. Chem. Soc.*, 1972, **94**, 1466–1475.

[13] M. C. R. Cockett, H. Ozeki, K. Okuyama and K. Kimura, *J. Chem. Phys.*, 1993, **98**, 7763–7772.

[14] Z. Liu, J. S. A. Ishibashi, C. Darrigan, A. Dargelos, A. Chrostowska, B. Li, M. Vasiliu, D. A. Dixon and S.-Y. Liu, *J. Am. Chem. Soc.*, 2017, **139**, 6082–6085.
